# Amyloid and Hydrogel Formation of a Peptide Sequence from a Coronavirus Spike Protein

**DOI:** 10.1021/acsnano.1c10658

**Published:** 2022-01-04

**Authors:** Valeria Castelletto, Ian W. Hamley

**Affiliations:** † Department of Chemistry, University of Reading, Reading RG6 6AD, United Kingdom

**Keywords:** amyloid, coronavirus, spike protein, nanotapes, aggregation, hydrogels

## Abstract

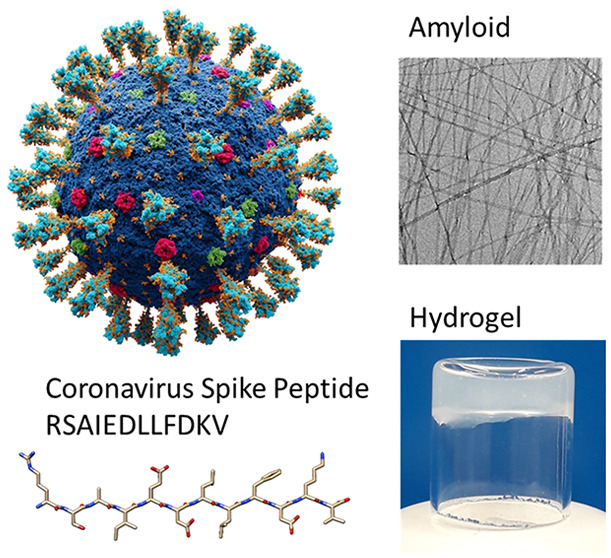

We demonstrate that a conserved coronavirus spike protein peptide forms amyloid structures, differing from the native helical conformation and not predicted by amyloid aggregation algorithms. We investigate the conformation and aggregation of peptide RSAIEDLLFDKV, which is a sequence common to many animal and human coronavirus spike proteins. This sequence is part of a native α-helical S2 glycoprotein domain, close to and partly spanning the fusion sequence. This peptide aggregates into β-sheet amyloid nanotape structures close to the calculated pI = 4.2, but forms disordered monomers at high and low pH. The β-sheet conformation revealed by FTIR and circular dichroism (CD) spectroscopy leads to peptide nanotape structures, imaged using transmission electron microscopy (TEM) and probed by small-angle X-ray scattering (SAXS). The nanotapes comprise arginine-coated bilayers. A Congo red dye UV–vis assay is used to probe the aggregation of the peptide into amyloid structures, which enabled the determination of a critical aggregation concentration (CAC). This peptide also forms hydrogels under precisely defined conditions of pH and concentration, the rheological properties of which were probed. The observation of amyloid formation by a coronavirus spike has relevance to the stability of the spike protein conformation (or its destabilization *via* pH change), and the peptide may have potential utility as a functional material. Hydrogels formed by coronavirus peptides may also be of future interest in the development of slow-release systems, among other applications.

## Introduction

**Figure 1:**
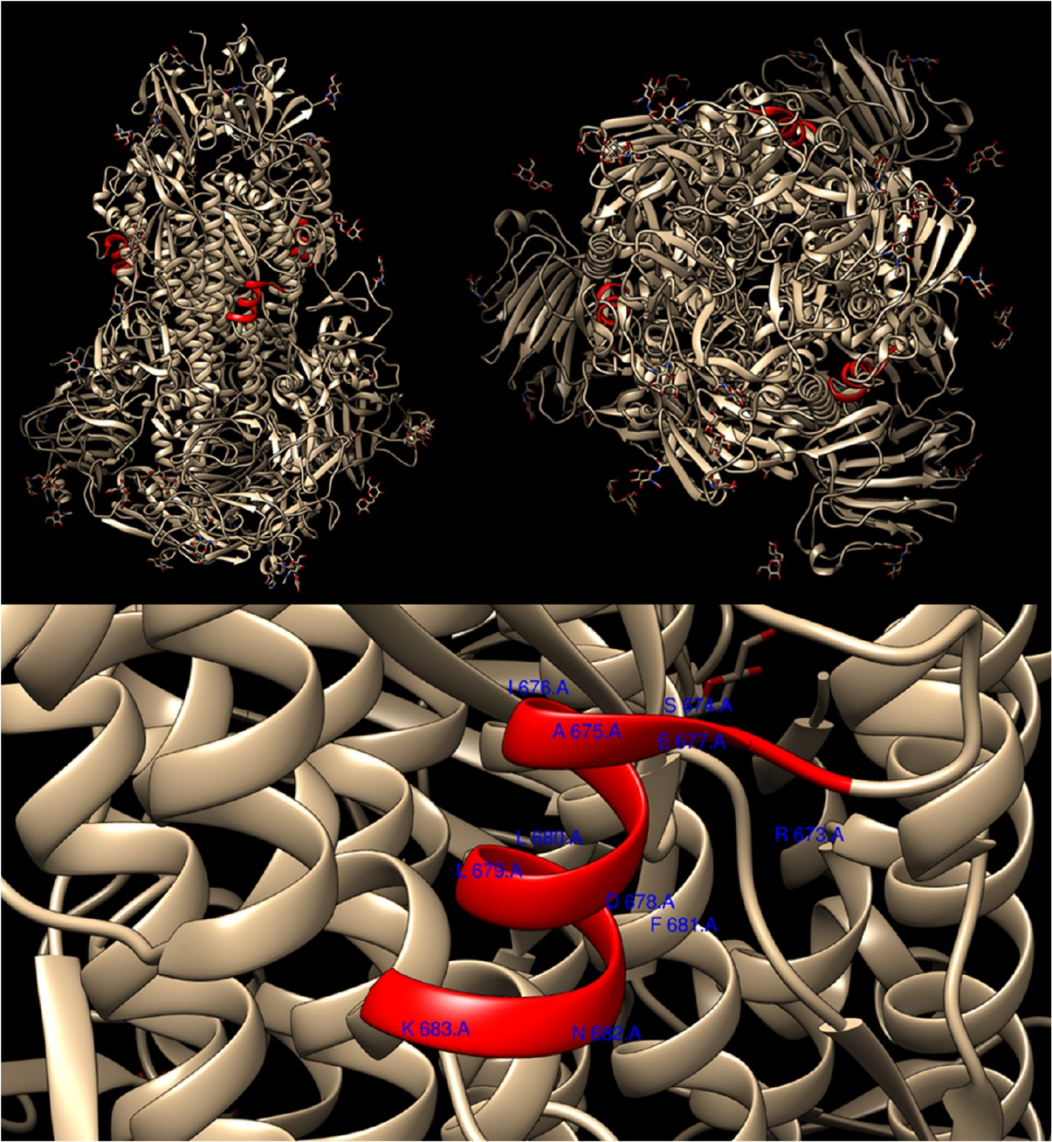
Structure of the porcine deltacoronavirus spike protein, obtained from high resolution cryo-EM.^3,4^ The sequence RSAIEDLLFNKV is highlighted (in red); this is the closest sequence to RSAIEDLLFDKV for which a pdb file could be obtained. The spike has a trimeric structure. Top, side and top views; bottom, enlargement of RSAIEDLLFNKV region with residue numbers for the A chain. This clearly lies in a surface coil sequence.

The COVID-19 pandemic has stimulated immense research activity into the SARS-CoV-2 coronavirus spike protein and its fusion process with human cells. The sequence of this protein has been determined and key regions involved in the binding of the spike glycoprotein have been identified, in the context of determining potential targets for therapeutic intervention. The sequence RSAIEDLLFDKV is found in many coronaviruses including the human common cold coronavirus spike protein as well as other coronavirus spike proteins from other animals.^1^ It immediately follows the second (S2’) cleavage site originally mapped in SARS-CoV and MERS-CoV,^2^ and later closely related sequences were identified in SARS-CoV-2.^1^ The structure of the single-residue substitution sequence RSAIEDLLFNKV has been determined by cryo-electron microscopy (this is the closest sequence match to RSAIEDLLFDKV, for which a high resolution protein structure is available). This reveals that this sequence (part of the porcine deltacoronavirus spike protein) is located close to, and partly overlaps, the fusion sequence by which the viral membrane and host cells fuse.^3,4^Figure 1 shows the spike protein structure, and a zoom into the RSAIEDLLFNKV region, which is clearly within a coil sequence in the native protein. A BLAST protein sequence alignment server search^5,6^ for RSAIEDLLFDKV (results shown in Table S1) revealed 106 results for corona virus spike protein sequences overlaps (with 75%–100% similarity) for this sequence, including 23 cases with 100% overlap with sequences for a variety of human and animal coronaviruses including MERS and bat coronavirus. This sequence and homologues form part of conserved domain^7^ cd 22369 (NCBI), which comprises SD-1 and SD-2 subdomains, the S1/S2 cleavage region, and the S2 fusion subunit of the spike (S) protein from alpha coronaviruses including human coronaviruses, porcine coronaviruses, transmissible gastroenteritis virus, and porcine epidemic diarrhea virus, among others.^8^ Among the related sequences, KRSFIEDLLFNKV is also a well-conserved sequence in the region around one of the known cleavage sites of SARS viruses that are believed to be required for virus activation for cell entry.^9,10^

Here, we investigate the pH-dependent self-assembly and gelation of the coronavirus spike protein peptide RSAIEDLLFDKV, which is conserved across many coronaviruses including SARS-CoV, MERS, and others, with only substitutions for some viruses at positions 5, 10, and 11.^2^ This peptide contains two cationic residues (one arginine and one lysine) and anionic residues (one glutamic acid and two aspartic acids) and has an expected pI at pH 4.2 (calculated using Innovagen’s web calculator PepCalc^11^)

Amyloids are β-sheet fibril structures formed by peptides and misfolded proteins.^12−14^ They are implicated in conditions such as neurodegenerative disease.^15−18^ Amyloids may be formed naturally and can have useful functional or bioactive roles *in vivo*, and recently, many examples of native peptides that can form amyloids have been uncovered.^13,14,19−22^ However, to the best of our knowledge, amyloid formation by SARS-CoV-2 peptides or proteins has not previously been reported, although it has recently been suggested that the SARS-CoV-2 spike receptor-binding domain may seed amyloid formation due to its heparin-binding properties, with this process being associated with the seeding of pathological amyloids.^23^ Here, we demonstrate the formation of β-sheet fibril structures by the conserved coronavirus spike protein sequence, RSAIEDLLFDKV. This is unexpected since closely related sequences lie in coil regions of the spike S2 domain surface (Figure 1), and, in addition, as discussed below, amyloid aggregation tendency algorithms incorrectly predict that this sequence will not form amyloid structures. We also show that this peptide exhibits pH-dependent self-assembly and hydrogelation behavior.

## Results and Discussion

**Figure 2:**
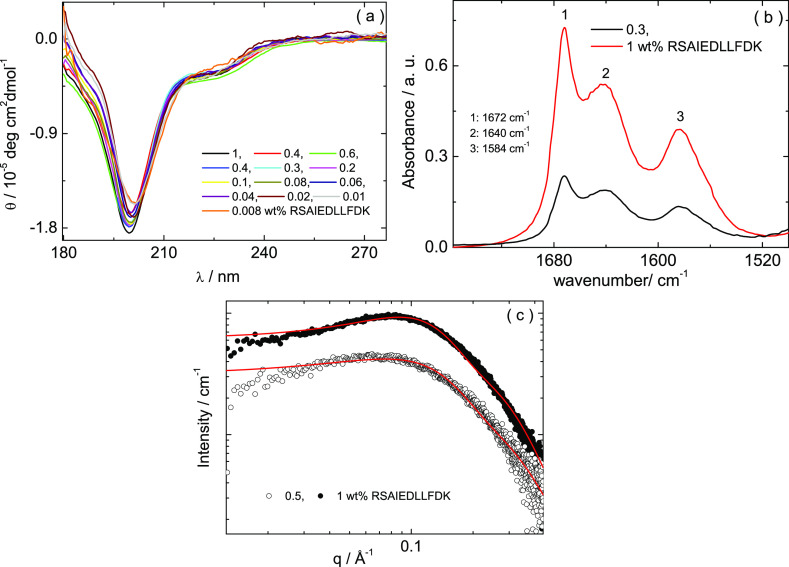
(a) CD, (b) FTIR, and (b) SAXS (open symbols, measured; red lines, fitted form factor profiles) for solutions in water, at the concentrations indicated.

Although many natural peptides and proteins have been shown to form amyloid, as of yet this has not been reported for coronavirus fragments. We investigated possible amyloid formation by the coronavirus spike protein peptide RSAIEDLLFDK. The secondary structure was first probed in aqueous solutions at native pH (the pH for 1 wt % peptide solution in water is pH 3) using CD and FTIR spectroscopy, while SAXS provided an excellent method to probe the self-assembled nanostructure (Figure 2). Figure 2a shows the CD spectra for the peptide for a series of concentrations. The shape of the CD spectra, characterized by a minimum at 192 nm, corresponds to a disordered (“random coil”) secondary structure.^35−37^ The disordered secondary structure remains constant for all concentrations studied, as shown in Figure 2a. Figure 2b shows the FTIR spectra measured for 0.3 and 1 wt % peptide. Both spectra are characterized by FTIR bands at 1672, 1640, and 1584 cm^–1^. The band at 1672 cm^–1^ corresponds to the FTIR signal of the trifluoroacetate (TFA) counterions (from the peptide salt).^38−40^ The bands at 1640 and 1584 cm^–1^ correspond to a disordered secondary structure^41−43^ and to amino acid side chain adsorption of the aspartate (D) residue, respectively.^43,44^Figure 2c shows the SAXS curves measured for 0.5 and 1 wt % peptide. SAXS curves have been fitted according using a form factor for monomers represented as Gaussian coils, along with a structure factor contribution required to fit the maximum in the intensity observed as a low *q* peak. This was represented as a Gaussian function, which represents a distribution of interparticle separations. The parameters extracted from the fitting in Figure 2c are listed in Table S2. The radius of gyration was determined to be *R*_g_ = 7 Å and 7.3 Å at 1 and 0.5 wt % peptide, respectively. The extended length of the RSAIEDLLFDK peptide is expected to be approximately 11 × 3.2 = 35.2 Å, assuming an extended β-sheet structure.^45^ The radii determined from SAXS thus indicate a compact conformation, consistent with the disordered secondary structure indicated by the CD and the FTIR spectra in Figure 2a,b. The results in Figure 2 clearly show that the peptide has a disordered secondary structure and is present as monomers in aqueous solutions.

Amyloid formation by peptides can be induced by pH adjustment, which changes the net charge on peptide molecules and hence can facilitate aggregation into intermolecular β-sheet structures.^14,18,46^ We reasoned that increasing the pH could induce the aggregation of the peptide, in particular close to the isoelectric point (calculated to be pH 4.2) since at this pH the net charge on the molecules will be zero, potentially enabling intermolecular association into β-sheet amyloid structures. Figure S1a shows the concentration of the NaOH solutions used to prepare 1 wt % RSAIEDLLFDK solutions at pH 4–12 and a curve plot of NaOH concentrations used to obtain different peptide concentrations.

**Figure 3:**
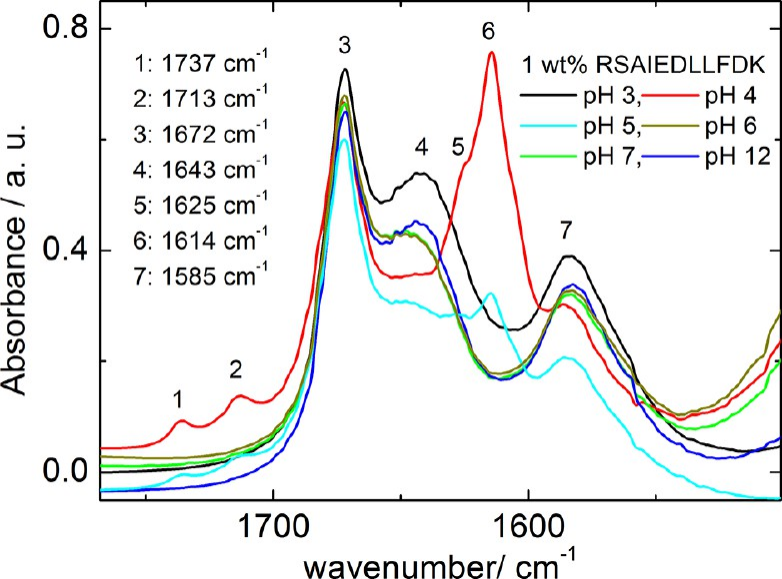
FTIR spectra for 1 wt % peptide as a function of pH (pH 3–12; solutions dissolved in pure D_2_O are native, with pH 3). The spectrum for the pH 3 sample is the same as that displayed in Figure 2b.

To characterize conformation and self-assembly at pH values higher than native we used FTIR and CD spectroscopy. Figure 3 shows the FTIR spectra measured for 1 wt % RSAIEDLLFDKV as a function of the pH, in the range pH 3–12. The FTIR spectra at pH 3, 6, 7, and 11 share the same features, and correspond to a disordered secondary structure, as discussed above for pH 3. The FTIR data for pH 4 and 5 are characterized by bands at 1737, 1713, 1672, 1614, and 1585 cm^–1^. The FTIR band at 1614 cm^–1^ corresponds to a β-sheet structure.^41−44^ FTIR bands at 1737 and 1713 cm^–1^ are stretching vibrations of the functional group COOH and from the C=O stretch of a protonated carboxyl side chain, in glutamic acid (E) or aspartic acid (D) residues or the C terminus.^42^ As mentioned above, the FTIR band at 1584 cm^–1^ corresponds to a side chain adsorption of the aspartate (D) residue.^42,44^Figure S2 presents the CD spectra for 1 wt % RSAIEDLLFDK as a function of the pH. The CD data at pH 3, 7, and 12 correspond to a disordered secondary structure, as the spectra are characterized by a minimum at 195 nm. The CD data at pH 5 and 6 is characterized by a maximum at 210 nm and a minimum at 220 nm, while the CD spectrum at pH 4 is characterized by a maximum at 190 nm and a minimum at 210 nm. The CD data shows the development of β-sheet secondary structure at pH 4, with β-sheet content also present at pH 5–6. However, a disordered structure is present again at a higher pH (pH 7 and 12). These results show that β-sheet secondary structure is induced close to the estimated pI of the peptide. Under these conditions, the charge is close to zero so that there is no electrostatic repulsion between peptide molecules, which are thus able to form intermolecular hydrogen bonds in β-sheet structures.

**Figure 4:**
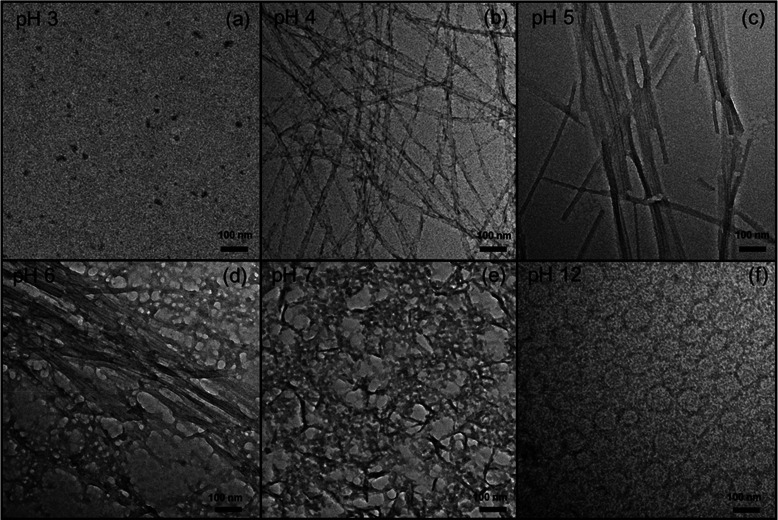
TEM images for 1 wt % RSAIEDLLFDKV solutions dissolved in (a) water (native; pH 3) or in NaOH aqueous solutions adjusted to give pH (b) 4, (c) 5, (d) 6, (e) 7, and (f) 12.

The potential presence of amyloid-like fibrillar structures was probed using transmission electron microscopy (TEM). Figure 4 shows TEM images measured for peptide solutions with pH 3–12. The TEM for the sample dissolved in water (Figure 4a) shows the absence of fibrillar structures; only a small number of globular structures were noted, consistent with the presence of disordered peptide monomers and possibly oligomers (not detected by SAXS) in solution. In contrast, the TEM images for 1 wt % peptide at pH 4 (Figure 4b) clearly show the presence of extended amyloid structures, in particular nanotapes. TEM images for a sample at pH 5 show that the peptide self-assembles into wide nanotapes, up to 100 nm thick, which in some areas unbundle into ∼20 nm thick nanotapes (Figure 4c). TEM images at pH 6 (Figure 4d) show a coexistence of nanotapes, with a morphology very similar to that observed at pH 5, with globular aggregates 30–40 nm in size. At a higher pH (pH 7 and 12), the TEM images (Figure 4e,f) show mesh-like structures, under conditions where CD indicates that the peptide molecules have a disordered conformation. The absence of extended amyloid structures is clear.

**Figure 5:**
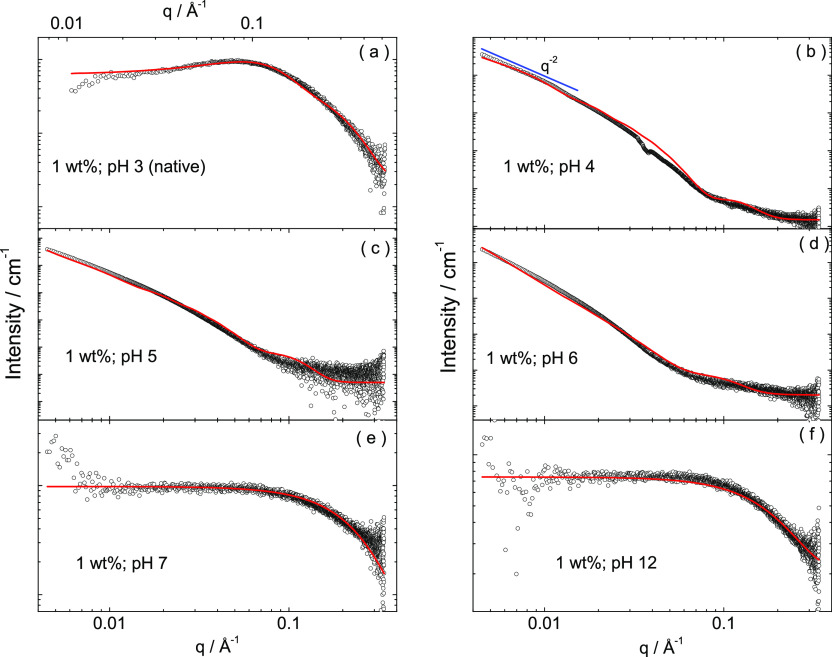
Measured SAXS data (open symbols) with fitted form factor profiles (red lines) for the same 1 wt % RSAIEDLLFDK samples at the pH values indicated. The limiting slope of *I*(*q*) ∼ *q*^–2^ shown (blue in b) confirms the presence of planar structures, here, nanotapes.

Small-angle X-ray scattering (SAXS) measurements were performed to provide additional quantitative information on the nanostructure in solution.^29^ SAXS data for 1 wt % samples at pH 3–12 are shown in Figure 5, along with fitted analytical form factor models described in the Experimental Section. The parameters extracted from the fitted form factor profiles (shown as red lines in Figure 5) are listed in Table S3. The data at pH 4−6 could be fitted using a model used for peptide nanotapes. The data show a limiting slope *I*(*q*) ∼ *q*^–2^ at a low *q*, which is characteristic of planar structures such as nanotapes,^29^ consistent with the TEM images. The intensity profiles were fitted to a model for peptide nanotapes based on a bilayer packing with a dense core and a diffuse electron density at the tape surfaces.^30−34^ Electron density profiles (across the nanotape thickness) were calculated using the parameters in Table S3, with a representative example being shown in Figure S3. This indicates the presence of a dense core of approximately 40 Å in extent, with diffuse outer layers. Considering that the extended length of RSAIEDLLFDK peptide in a β-strand conformation is estimated to be 40.8 Å, this is based on a translation per residue in an antiparallel β-sheet of 3.4 Å.^45^ Therefore, the molecules are interdigitated,^47^ leading to the proposed models shown in Figure S4. It is likely that the hydrophobic core comprises the LLF residues (in particular, stacking of the phenylalanine residues is evident in Figure S4) while the charged residues are present on each side of the bilayer structure, forming extended outer layers. It can be noted that unfavorable electrostatic interactions between terminal lysine or arginine residues are likely to favor an antiparallel arrangement as shown, with salt bridging also possible between oppositely charged residues such as K and D. The SAXS form factor permits a clear determination of the bilayer thickness, *t*, but the width of the nanotapes (ca. 175 Å from the TEM image analysis) is too large to be determined from the SAXS data (the bilayer width, represented as diameter *D* is set to a large value, changing which simply acts as a scale factor of the form factor if *D* ≫ *t*). The SAXS data also indicates that the nanotapes predominantly comprise single bilayers since there is no evidence for any Bragg peaks from a multilayer structure. This is shown in the proposed model structures in Figure S4. The SAXS data for samples at pH 7 and 12 can be fitted using form factors for monomers (Gaussian coils) similar to the data at pH 3 (Tables S2 and S3).

**Figure 6:**
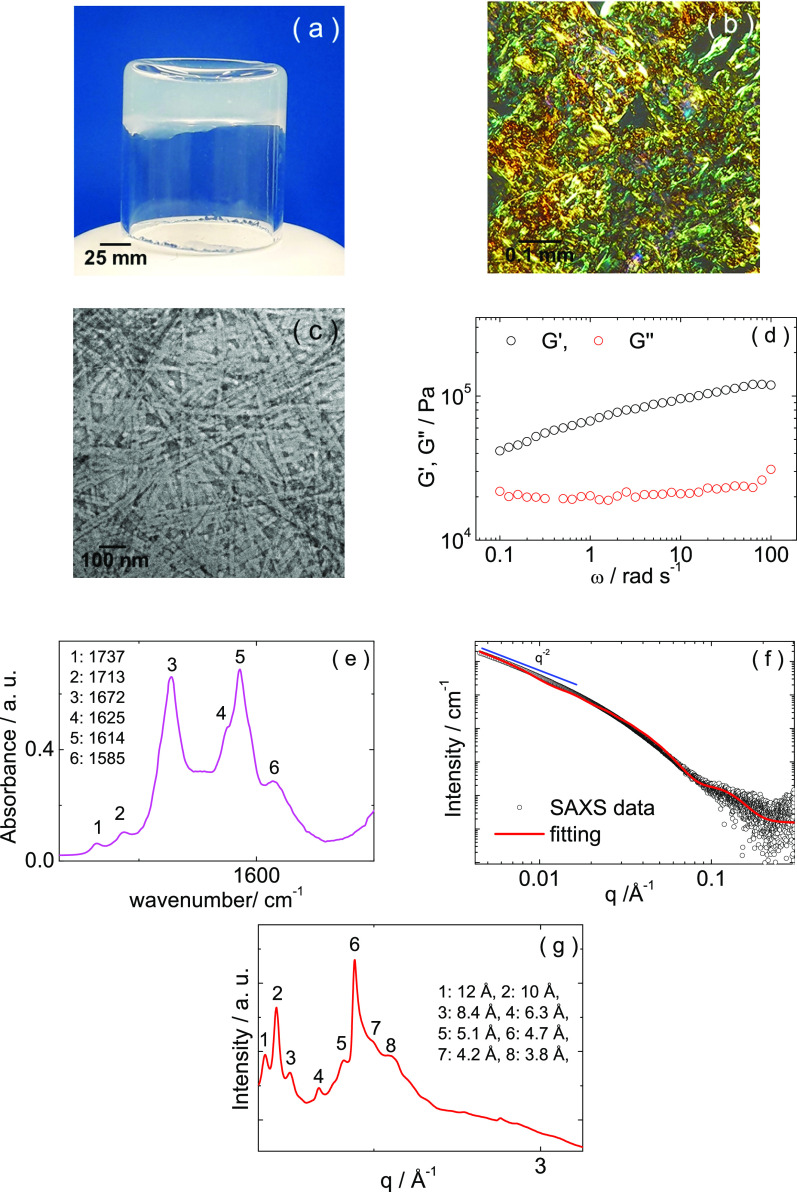
Gel formation for 1 wt % RSAIEDLLFDK at pH 4.4: (a) image of gel in vial, (b) polarized optical microscopy (POM) image showing Congo red birefringence, (c) TEM, (d) rheology (frequency sweep of shear moduli), (e) FTIR, (f) SAXS (showing limiting low *q* slope in blue), and (g) XRD.

Peptide RSAIEDLLFDK is able to form a hydrogel under precisely defined conditions. All the samples discussed above are liquid at 1 wt % peptide; however, we found that a 1 wt % peptide sample at pH 4.4 forms a gel. Figure 6a shows that a 1 wt % peptide gel at pH 4.4 does not flow under tube inversion but rather forms a cloudy gel. The apple-green birefringence of the peptide gel stained with Congo red (Figure 6b) together with TEM images (Figure 6c) reveal that the hydrogel comprises a tight network of 20–50 nm wide peptide nanotapes. The viscoelastic response of the gel was studied by rheology. The linear regime was first identified by strain sweep experiments (Figure S5). A frequency sweep of shear moduli is shown in Figure 6d. The elastic modulus *G*′ > *G*′′ (the shear loss modulus) and *G*′ is weakly dependent on frequency and reaches a value >10^5^ Pa at a high frequency (ω = 100 rad s^–1^). These are all characteristic rheological signatures of gels, and the high frequency value of *G*′ is higher than that observed for many peptide hydrogels.^48^ The FTIR spectrum shown in Figure 6e is very similar to that discussed for the solutions of 1 wt % RSAIEDLLFDK at pH 4 and 5 and is consistent with a β-sheet secondary structure. The SAXS data was again fitted using a model form factor of Gaussian bilayers (Figure 6f). The fitting parameters are listed in Table S3. The XRD data is characterized by strong diffraction peaks at 10 and 4.7 Å, consistent with an amyloid cross-β diffraction pattern.^12,49−51^ The other weaker peaks can also be ascribed to β-sheet features.^49,52−57^

**Figure 7:**
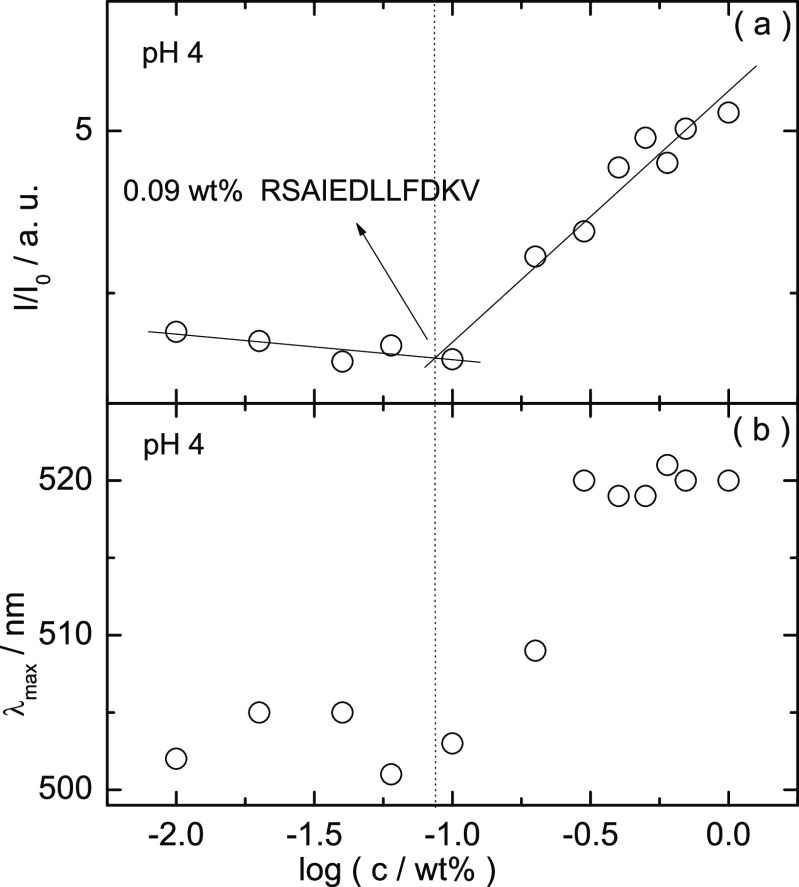
Congo red UV–vis assay: (a) intensity and (b) emission wavelength as a function of the peptide concentration (pH 4) (*I* and *I*_0_ are the intensities for samples with and without peptide, respectively).

Having determined the lowest pH value for β-sheet nanotape formation, the potential presence of a critical aggregation concentration (CAC) at this pH value (pH 4) was assayed using Congo red, a stain commonly used to probe the presence of amyloid fibers in solution.^12,24−26^ In contrast to its conventional use to stain amyloid, leading to green birefrigent textures in the polarized optical microscope, we employed a quantitative assay, measuring the UV–vis spectrum as a function of concentration and monitoring the peak position and absorbance. Figure S6 shows the UV–vis spectra for peptide solutions containing 0.015 wt % Congo red (the spectrum for a native solution is also displayed for comparison). Figure S6 shows that the maximum in the UV–vis spectra of 0.015 wt % Congo red become more intense upon increasing the concentration of peptide at pH 4. This effect is not observed (Figure S6) for the native pH solution, because there is no amyloid formation. Figure 7 shows a plot of the maximal peak intensity and position in the spectra as a function of the concentration at pH 4. The data in Figure 7 show a break point in the relative intensity and peak position at the CAC, indicating that amyloid structures are present in solution at concentrations higher than 0.09 ± 0.02 wt % peptide at pH 4. Polarized optical microscopy (POM) reveals that solutions with 0.4–1 wt % RSAIEDLLFDK at pH 4 stained with Congo red show a strong apple-green birefringence under crossed polarizers (Figure S7), in good agreement with the results from the analysis of the corresponding UV–vis spectra shown in Figure 7.

Figure S8 shows FTIR spectra as a function of the concentration at pH 4. The distinctive β-sheet features, discussed above for the data in Figure 2, are observed for 0.4–1 wt % peptide at pH 4. Lower concentrations did not give a good FTIR signal. Figure S9 shows the CD data as a function of the concentration at pH 4, which are also consistent with a β-sheet structure across this concentration range.

**Figure 8:**
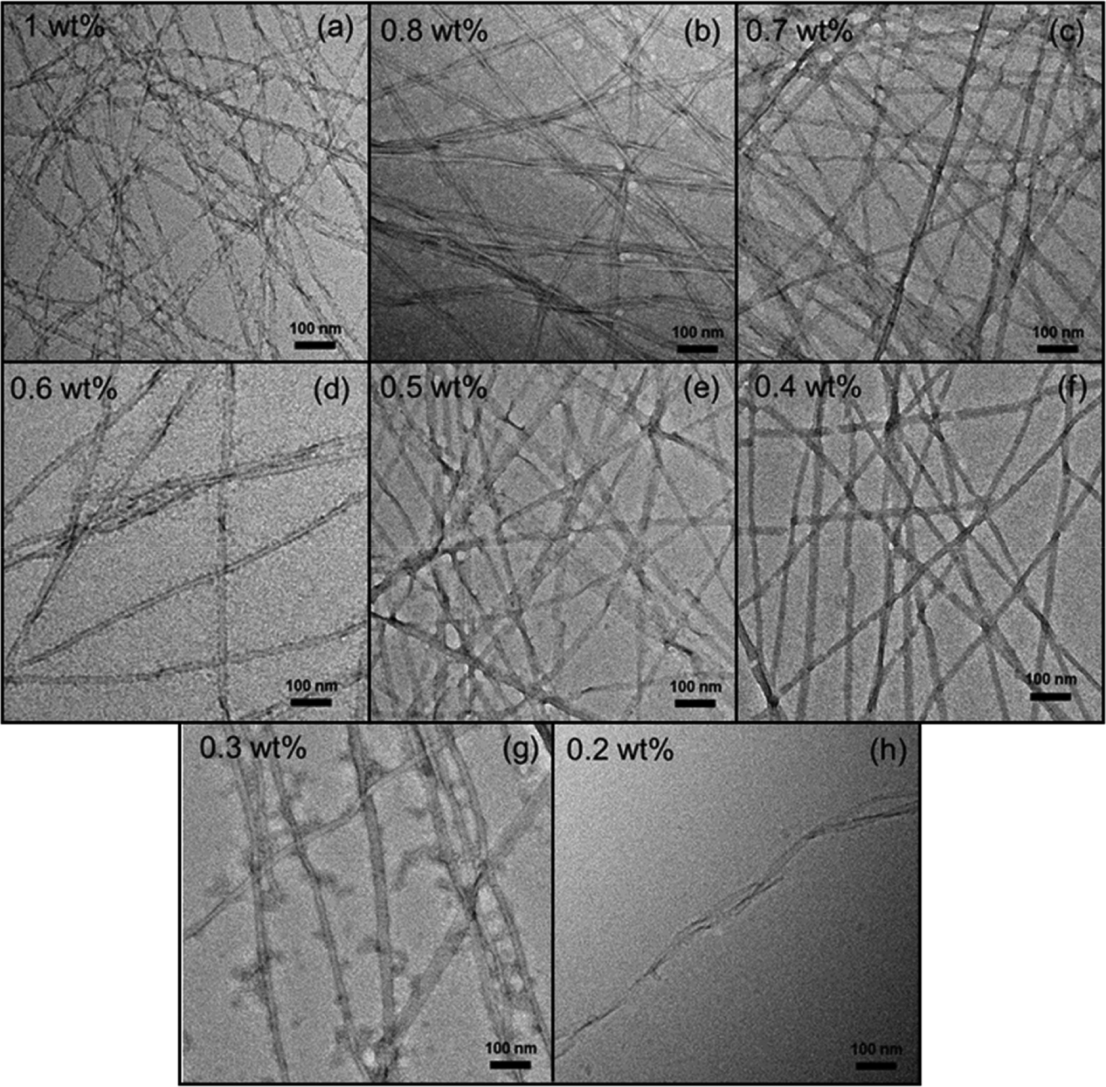
TEM images for (a) 1, (b) 0.8, (c) 0.7, (d) 0.6, (e) 0.5, (f) 0.4, (g) 0.3, and (h) 0.2 wt % RSAIEDLLFDKV (pH 4). The TEM image in (a) is the same as that displayed in Figure 4b, and it is shown for comparative purposes only.

**Figure 9:**
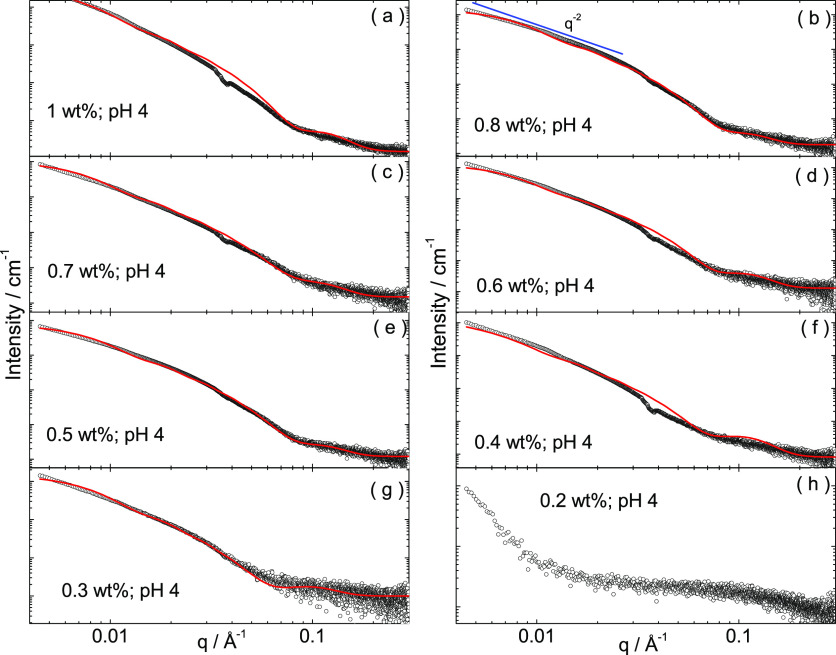
Measured SAXS data (open circles) and fitted form factors (red lines) for the same samples measured by TEM (images shown in Figure 8) at pH 4.

TEM images were obtained for samples containing 1–0.2 wt % RSAIEDLLFDKV (pH 4), and as shown in Figure 8 these reveal nanotape structures. The histograms corresponding to the distribution of nanotape widths measured from Figure 8 are displayed in Figure S10, together with the average nanotape widths as a function of the concentration, calculated from each histogram. The distribution of nanotape widths remains peaked at 15–25 nm, with the exception of the data for the 0.5 wt % sample, and the average nanotape width is 17 ± 4 nm across the concentration range studied (Figure S10b). It was also noted that some peptide nanotapes show grooves with a 9 nm periodicity. SAXS data was also acquired for the same samples imaged by TEM at pH 4. The SAXS intensity profiles shown in Figure 9a–g were fitted using the Gaussian bilayer model, as above. The SAXS curve for 0.2 wt % shows a contribution from a nanotape form factor at a low *q* and monomers at a high *q*. The parameters obtained from the fitting to the SAXS curves in Figure 9 are listed in Table S4. The data indicate relatively little change in the bilayer thickness, which ranges from 18 to 30 Å or in the width of the inner or outer Gaussians representing the electron density profile across the bilayers. The internal structure of the peptide nanotapes was also studied by XRD. Figure S11 shows the XRD spectra measured for stalks dried from peptide solutions containing 0.5 and 1 wt % solutions at pH 4. The XRD profiles show strong reflections at 9.8 and 4.7 Å, indicating a cross-β structure, as discussed above in the context of the data in Figure 6g. The minor peaks such as those at 3.8 Å are due to intra-β-sheet features, as indexed elsewhere.^49,52−57^

## Conclusions

The coronavirus spike protein fragment peptide RSAIEDLLFDKV, a sequence common to many animal and human coronaviruses, was shown to form amyloid structures at pH values close to the isoelectric point. This is an unexpected result, because, as shown in Figure 1, very closely related sequences (single residue D10N substitution, the closest sequence for which a high resolution structure is available) lie in α-helical regions of the coronavirus spike protein S2 domain. In addition, this result is unanticipated since this sequence is predicted to have no β-sheet aggregation propensity at pH 4 using TANGO^58,59^ or CamSol.^60,61^

The formation of β-sheet amyloids was comprehensively established using FTIR and CD to probe the conformation and TEM, SAXS, and XRD to determine the nanostructure. Our data demonstrate that the peptide transitions from disordered monomers into β-sheet nanotapes when the pH is increased from 3 to 4 at a fixed concentration of peptide. The classical signatures of amyloid formation were clearly revealed by the combination of spectroscopic, scattering, and imaging methods employed. A critical aggregation concentration for amyloid formation was determined using a Congo red UV–vis absorbance assay, along with POM imaging of the associated birefringent textures. This method complements other techniques using fluorescence dyes such as ThT to detect amyloid formation.^12,26,51,62−64^ SAXS indicates that the nanotapes predominantly comprise single bilayers. According to our model, this is due to the sequestration of arginine at the nanotape surfaces. These arginine-coated nanotapes resemble the arginine-coated nanosheets previously reported for arginine-capped surfactant like peptides.^32,65^

The aggregation behavior of RSAIEDLLFDKV was found to be highly pH sensitive, since hydrogelation was observed at pH 4.4 but not pH 4. The hydrogel is formed from a β-sheet nanotape network and has a high value of the shear elastic modulus, with a *G*′ exceeding 10^5^ Pa at a high frequency. The pH at which hydrogel formation is observed was precisely determined (pH 4.4). It is suggested that this may be the real pI of the peptide (pI = 4.2 calculated), since gelation due to peptide nanotape network formation can occur at the isoelectric point.^66−68^ Future work could also explore whether a slow pH change (as opposed to the essentially instantaneous pH change effected by the addition of NaOH) can lead to more homogeneous or transparent gels, as demonstrated for short peptide derivatives, using the hydrolysis of urea by the enzyme urease producing ammonia, which results in a slow increase in the pH.^69,70^

Our findings should stimulate future work to probe potential amyloid formation by coronavirus-related peptides and proteins. We suggest that the initiation of amyloid formation by such sequences by pH change may disrupt interactions with cell receptors, although the presence of counterions *in vivo* should be considered in future studies of aggregation. It will also be of interest to investigate amyloid formation triggered by other means (specific chemical cues for instance) as a potentially useful “denaturing” tool. Apart from understanding the folding states of coronavirus peptides and proteins, with potential relevance to therapeutics, our work also introduces a natural (bioderived) sequence that forms amyloid structures with possible functional amyloid properties in nanomaterials applications,^20,21^ for instance for filtration, as scaffolds for inorganic materials or as structural elements. The hydrogel formed by RSAIEDLLFDKV may also be valuable as a pH-triggered gel and/or for slow release applications.

## Experimental Section

### Materials

Peptide RSAIEDLLFDKV was supplied by Peptide Synthetics (Peptide Protein Research Limited), Fareham, UK as a TFA salt. The molar mass by electrospray mass spectrometry is 1405.595 g mol^–1^ and the purity is >95%, determined by high performance liquid chromatography (HPLC) in a gradient HPLC using an acetonitrile (0.1% TFA)/water (0.1% TFA) gradient with a Kinetex 2.6u XB-C18 100A column. A set of samples was prepared by mixing weighed amounts of peptide and pure Milli-Q water. Where indicated, the pH of samples was increased to pH 4–12 by mixing weighed amounts of peptide with NaOH solutions. Figure S1a shows the concentration of NaOH solutions used to prepare 1 wt % peptide solutions at pH 4–12, while Figure S1b shows the concentration of NaOH solutions used to prepare 0.2–1 wt % peptide sols at pH 4. In the following, samples prepared using pure water as a solvent will be referred as “native”.

### UV–Visible Spectroscopy

Congo red is a dye that stains amyloid in solution, giving a characteristic green birefringence.^12,24−26^ In contrast to this use of Congo red, we measured the UV–vis spectra of peptide samples stained with the dye to quantitatively determine the onset of peptide fibril formation (at the CAC) with increasing concentration, at pH 4. UV–vis spectra were measured using a Nanodrop instrument. For each experiment, an aliquot of 95 μL of peptide solution was stained by injecting 5 μL of 0.3 wt % Congo red, to obtain a final concentration of 0.015 wt % Congo red. The UV–vis spectra were measured for wavelengths in the range 190–850 nm. Results from the Congo red assays were analyzed as *I*/*I*_0_ and λ_em_ vs log(c) (*I* and *I*_0_, maximum intensity of emission for Congo red solutions with and without peptide, respectively; λ_em_, emission wavelength for Congo red solutions with peptide).

### Circular Dichroism (CD) Spectroscopy

CD spectra were recorded using a Chirascan spectropolarimeter (Applied Photophysics, Leatherhead, UK). Solutions were placed between parallel plates (0.01 mm path length). Spectra were measured with a 0.5 nm step, 1 nm bandwidth, and 1 s collection time per step. The CD signal from the water background was subtracted from the CD data of the sample solutions.

### Fourier Transform Infrared (FTIR) Spectroscopy

Spectra were recorded using a Thermo-Scientific Nicolet iS5 instrument equipped with a DTGS detector, with a Specac Pearl liquid cell (sample contained between fixed CaF_2_ plates). Spectra (1 wt % sample in H_2_O) were scanned 128 times over the range 900–4000 cm^–1^.

### Small-Angle X-ray Scattering (SAXS)

Synchrotron SAXS experiments on solutions were performed using BioSAXS robots on beamline BM29 at the ESRF (Grenoble, France) and on beamline B21 (Diamond Light Source Ltd.). On beamline BM29, a few microliters of samples were injected *via* an automated sample exchanger at a slow and very reproducible rate into a quartz capillary (1.8 nm internal diameter) in the X-ray beam. The quartz capillary was enclosed in a vacuum chamber in order to avoid parasitic scattering. After the sample was injected in the capillary and reached the X-ray beam, the flow was stopped during the SAXS data acquisition. The *q* range was set to 0.004–0.4 Å^–1^, with λ = 1.03 Å. The images were captured using a Pilatus 1 M detector. Data processing (background subtraction, radial averaging) was performed using dedicated beamline software ISPyB. On beamline B21, solutions were loaded into the 96-well plate of an EMBL BioSAXS robot and then injected *via* an automated sample exchanger into a quartz capillary (1.8 mm internal diameter) in the X-ray beam. The quartz capillary was enclosed in a vacuum chamber. The flow of the sample through the capillary was continuous during the SAXS data acquisition. The peptide gel was placed in a custom-designed sample holder for gels,^27^ which was placed inside a vacuum chamber, oriented perpendicular to the path of the incident X-ray beam. Beamline B21 operated with a fixed camera length (3.9 m) and a fixed wavelength (λ = 1.00 Å). The images were captured using a Pilatus 2 M detector. Data processing (background subtraction, radial averaging) was performed using the dedicated beamline software ScÅtter.

### SAXS Models

The SAXS curves were fitted using a form factor for a Gaussian chain^28,29^ to describe nonassembled peptide molecules or a form factor for Gaussian bilayers^30^ to represent the electron density variation across the bilayers, as used previously by our group for peptide-based nanotape assemblies.^31−33^ A Gaussian function was used as an approximation to describe the structure factor.^29^ The fitting parameters for the Gaussian chain form factor are the radius of gyration, *R*_g_, and the scattering length of the solvent, ρ_so_. The Gaussian membrane form factor was originally formulated for a lipid bilayer. The model assumes an electron density profile comprising Gaussian functions for the head groups on either side of the membrane and another Gaussian for the hydrocarbon chain interior. The midpoint of the bilayer is defined as *z* = 0 = *z*_C_.^30^ The model assumes a distribution of interhead group thickness 2*z*_H_, with an associated degree of polydispersity Δ_2z_H__. The fitting parameters of the model are the electron densities of the headgroups, η_H_, the layer thickness, 2*z*_H_ (and its polydispersity), the electron density of the hydrocarbon chains, η_H_, the width of the Gaussian peak at *z*_H_, and the width σ_C_ of the Gaussian peak at *z*_C_. All fitting was done using the software SASfit.^28^ The fitting parameters for the Gaussian amplitude structure factor are the amplitude, *A*, the position, *q*_0_, the width, *w*, and the background, *C*.

### Transmission Electron Microscopy (TEM)

TEM imaging was performed using a JEOL 2100Plus TEM microscope operated at 200 kV. For liquid samples, a drop of solution was placed on Cu grids coated with a carbon film (Agar Scientific), stained with 1 wt % uranyl acetate acid (u.a.; Sigma-Aldrich), and dried. Excess peptide and staining solutions were blotted using filter paper. TEM grids for the peptide gel were prepared by placing a small amount of gel on a TEM grid using a spatula. The excess of gel was removed by blowing compressed air. The residual gel on the surface of the TEM grid was stained using 1 wt % u.a. and dried.

### Rheology

Dynamic shear moduli were measured using a controlled stress AR-2000 rheometer from TA Instruments. A cone and plate geometry (20 mm diameter, 1° angle) was used for the peptide gel (1 wt % peptide at pH 4.4). Stress sweeps were performed first to determine the linear viscoelastic regime. Frequency sweeps were performed using a constant stress σ = 30 Pa for angular frequencies in the range ω = 0.1–100 rad s^–1^.

### Polarized Optical Microscopy (POM)

A drop of sample was placed on a microscope slide and stained with a solution containing 0.3 wt % Congo red. The stained sample was covered with a microscope coverslip and observed through the crossed polarizers of a Olympus BX41 polarized microscope. Images were captured using a Canon G2 digital camera fitted to the microscope.

### X-ray Diffraction (XRD)

Measurements were performed on stalks prepared by drying a drop of solution suspended between the ends of wax-coated capillaries. The stalks were mounted onto a four axis goniometer of an Oxford Diffraction Gemini Ultra instrument. The sample–detector distance was 44 mm. The X-ray wavelength λ = 1.54 Å was used to calculate the scattering vector *q* = 4π sin θ/λ (2θ: scattering angle). The detector was a Sapphire CCD.

## Supporting Information Available

Tables of BLAST results for RSAIEDLLFDKV and parameters extracted from the fitting of SAXS data, and figures of concentration of NaOH solutions used as a solvent to obtain different pH solutions, concentration of NaOH solutions used to obtain different peptide concentrations, CD spectra, electron density profile, two possible models for molecular packing in the arginine-coated bilayer nanotapes, shear moduli versus stress, UV–vis spectra, polarized optical microscopy images, FTIR spectra, histograms showing the distribution of fibre widths measured from TEM images, average fibre width as a function of the concentration, and XRD data (PDF)
