# Supplementary material for: Amyloid and Hydrogel Formation of a Peptide Sequence from a Coronavirus Spike Protein
Source: ACS Nano. 2022 Jan 4;16(2):1857–67. doi: 10.1021/acsnano.1c10658 (PMC8867915; doi:10.1021/acsnano.1c10658)
Supplement: Supplementary file 1 — nn1c10658_si_001.pdf [file nn1c10658_si_001.pdf]

Supplementary Information

Amyloid and Hydrogel Formation of a Peptide Sequence from a Coronavirus Spike Protein

Valeria Castelletto,[†,*] and Ian W. Hamley[†,*]

[†]Department of Chemistry, University of Reading. Reading RG6 6AD, United Kingdom.

Table S1. BLAST results for RSAIEDLLFDKV from https://www.uniprot.org/blast/uniprot/B202110065BF3C56A578D7D6DFD1FC81EE5DA7730015C35N?sort=identity, Oct 13 2021)[a]

**Entry**	**Info**[b]	**Organism**
H9TEX4	E-value: 0.0017; Score: 89; Ident.: 100.0%	Canine coronavirus
Q7T6T3	E-value: 0.0017; Score: 89; Ident.: 100.0%	Canine coronavirus (strain BGF10) (CCoV) (Canine enteric coronavirus)
Q65984	E-value: 0.0017; Score: 89; Ident.: 100.0%	Canine coronavirus (strain K378) (CCoV) (Canine enteric coronavirus)
P36300	E-value: 0.0017; Score: 89; Ident.: 100.0%	Canine coronavirus (strain Insavc-1) (CCoV) (Canine enteric coronavirus)
Q01977	E-value: 0.0017; Score: 89; Ident.: 100.0%	Porcine transmissible gastroenteritis coronavirus (strain NEB72-rt) (TGEV)
P07946	E-value: 0.0017; Score: 89; Ident.: 100.0%	Porcine transmissible gastroenteritis coronavirus (strain Purdue) (TGEV)
Q02385	E-value: 0.0017; Score: 89; Ident.: 100.0%	Murine coronavirus (strain JHMV / variant CL-2) (MHV) (Murine hepatitis virus)
P22432	E-value: 0.0017; Score: 89; Ident.: 100.0%	Murine coronavirus (strain 4) (MHV-4) (Murine hepatitis virus)
C0KYU0	E-value: 0.0017; Score: 89; Ident.: 100.0%	Murine coronavirus repA59/RJHM
K9N5Q8	E-value: 0.0017; Score: 89; Ident.: 100.0%	Middle East respiratory syndrome-related coronavirus (isolate United Kingdom/H123990006/2012) (MERS-CoV) (Betacoronavirus England 1)
P36334	E-value: 0.0017; Score: 89; Ident.: 100.0%	Human coronavirus OC43 (HCoV-OC43)
R9UQ53	E-value: 0.0017; Score: 89; Ident.: 100.0%	Middle East respiratory syndrome-related coronavirus (MERS-CoV)
A3EX94	E-value: 0.0017; Score: 89; Ident.: 100.0%	Bat coronavirus HKU4 (BtCoV) (BtCoV/HKU4/2004)
Q0Q4F2	E-value: 0.0017; Score: 89; Ident.: 100.0%	Bat coronavirus 133/2005 (BtCoV) (BtCoV/133/2005)
Q8JSP8	E-value: 0.0017; Score: 89; Ident.: 100.0%	Porcine hemagglutinating encephalomyelitis virus (strain IAF-404) (HEV)

Q8BB25	E-value: 0.0017; Score: 89; Ident.: 100.0%	Porcine hemagglutinating encephalomyelitis virus (strain 67N) (HEV-67N)
P11224	E-value: 0.0017; Score: 89; Ident.: 100.0%	Murine coronavirus (strain A59) (MHV-A59) (Murine hepatitis virus)
Q9J3E7	E-value: 0.0017; Score: 89; Ident.: 100.0%	Murine hepatitis virus
P11225	E-value: 0.0017; Score: 89; Ident.: 100.0%	Murine coronavirus (strain JHM) (MHV-JHM) (Murine hepatitis virus)
I7B987	E-value: 0.0017; Score: 89; Ident.: 100.0%	Murine coronavirus
H9BR00	E-value: 0.0017; Score: 89; Ident.: 100.0%	Sparrow coronavirus HKU17
A0A0S2ZWY7	E-value: 0.0017; Score: 89; Ident.: 100.0%	European turkey coronavirus 080385d
B6VDX8	E-value: 0.0017; Score: 89; Ident.: 100.0%	Thrush coronavirus HKU12 (isolate Grey-backed thrush/Hong Kong/HKU12-600/2007) (ThCoV-HKU12) (Thrush coronavirus HKU12-600)
A0A0K2RVL1	E-value: 0.013; Score: 83; Ident.: 91.7%	Equine coronavirus
A0A023Y9K3	E-value: 0.013; Score: 83; Ident.: 91.7%	BtVs-BetaCoV/SC2013
A0A060A825	E-value: 0.013; Score: 83; Ident.: 91.7%	Porcine coronavirus HKU15
A0A140ESF1	E-value: 0.013; Score: 83; Ident.: 91.7%	Porcine deltacoronavirus
P10033	E-value: 0.027; Score: 81; Ident.: 100.0%	Feline coronavirus (strain FIPV WSU-79/1146) (FCoV)
Q77NQ7	E-value: 0.038; Score: 80; Ident.: 91.7%	Murine coronavirus (strain 2) (MHV-2) (Murine hepatitis virus)
Q9IKD1	E-value: 0.038; Score: 80; Ident.: 91.7%	Rat coronavirus (strain 681) (RCV-SDAV) (Sialodacryoadenitis virus SDAV-681)
C6GHS2	E-value: 0.038; Score: 80; Ident.: 91.7%	Rat coronavirus Parker
U5LNM4	E-value: 0.038; Score: 80; Ident.: 91.7%	Betacoronavirus Erinaceus/VMC/DEU/2012
P33470	E-value: 0.076; Score: 78; Ident.: 91.7%	Porcine transmissible gastroenteritis coronavirus (strain Miller) (TGEV)

C6GHE6	E-value: 0.11; Score: 77; Ident.: 83.3%	Feline coronavirus UU15
A0A125R5A5	E-value: 0.11; Score: 77; Ident.: 83.3%	Feline coronavirus
E3W5J0	E-value: 0.11; Score: 77; Ident.: 83.3%	Feline coronavirus UU30
P18450	E-value: 0.11; Score: 77; Ident.: 91.7%	Porcine transmissible gastroenteritis coronavirus (strain FS772/70) (TGEV)
P25194	E-value: 0.11; Score: 77; Ident.: 91.7%	Bovine coronavirus (strain vaccine) (BCoV) (BCV)
P25193	E-value: 0.11; Score: 77; Ident.: 91.7%	Bovine coronavirus (strain Quebec) (BCoV) (BCV)
Q9QAQ8	E-value: 0.11; Score: 77; Ident.: 91.7%	Bovine coronavirus (strain OK-0514) (BCoV) (BCV)
P15777	E-value: 0.11; Score: 77; Ident.: 91.7%	Bovine coronavirus (strain Mebus) (BCoV) (BCV)
P25192	E-value: 0.11; Score: 77; Ident.: 91.7%	Bovine coronavirus (strain LY-138) (BCoV) (BCV)
Q8V436	E-value: 0.11; Score: 77; Ident.: 91.7%	Bovine coronavirus (strain 98TXSF-110-LUN) (BCoV-LUN) (BCV)
Q9QAR5	E-value: 0.11; Score: 77; Ident.: 91.7%	Bovine coronavirus (strain LSU-94LSS-051) (BCoV-LSU) (BCV)
P25191	E-value: 0.11; Score: 77; Ident.: 91.7%	Bovine coronavirus (strain L9) (BCoV) (BCV)
P25190	E-value: 0.11; Score: 77; Ident.: 91.7%	Bovine coronavirus (strain F15) (BCoV) (BCV)
Q91A26	E-value: 0.11; Score: 77; Ident.: 91.7%	Bovine coronavirus (strain 98TXSF-110-ENT) (BCoV-ENT) (BCV)
B7U2N3	E-value: 0.11; Score: 77; Ident.: 91.7%	Waterbuck coronavirus US/OH-WD358/1994
B7U2M2	E-value: 0.11; Score: 77; Ident.: 91.7%	Waterbuck coronavirus US/OH-WD358-GnC/1994
P24413	E-value: 0.11; Score: 77; Ident.: 91.7%	Porcine respiratory coronavirus (strain RM4) (PRCoV) (PRCV)
P27655	E-value: 0.11; Score: 77; Ident.: 91.7%	Porcine respiratory coronavirus (strain 86/137004 / isolate British) (PRCoV) (PRCV)

D9J204	E-value: 0.15; Score: 76; Ident.: 83.3%	Mink coronavirus strain WD1133
B1PHI8	E-value: 0.15; Score: 76; Ident.: 91.7%	Bat coronavirus 1B
A3EXD0	E-value: 0.15; Score: 76; Ident.: 83.3%	Bat coronavirus HKU5 (BtCoV) (BtCoV/HKU5/2004)
A0A0U1WHD7	E-value: 0.22; Score: 75; Ident.: 90.9%	BtMr-AlphaCoV/SAX2011
Q91AV1	E-value: 0.22; Score: 75; Ident.: 83.3%	Porcine epidemic diarrhea virus (strain CV777) (PEDV)
A0A0A7UZR7	E-value: 0.22; Score: 75; Ident.: 83.3%	Betacoronavirus HKU24
H9BR35	E-value: 0.22; Score: 75; Ident.: 90.9%	Common moorhen coronavirus HKU21
A0A0F6YS09	E-value: 0.22; Score: 75; Ident.: 91.7%	Duck coronavirus
A0A4D6FWE9	E-value: 0.22; Score: 75; Ident.: 91.7%	Canada goose coronavirus
B2BW33	E-value: 0.31; Score: 74; Ident.: 83.3%	Beluga whale coronavirus SW1
A0A088DJY6	E-value: 0.31; Score: 74; Ident.: 83.3%	Bat Hp-betacoronavirus/Zhejiang2013
A0A0U1WHB6	E-value: 0.43; Score: 73; Ident.: 90.9%	BtMf-AlphaCoV/GD2012
A0A0U1WHD9	E-value: 0.43; Score: 73; Ident.: 90.9%	BtMf-AlphaCoV/HeN2013
B1PHK2	E-value: 0.43; Score: 73; Ident.: 83.3%	Miniopterus bat coronavirus HKU8
Q0Q466	E-value: 0.43; Score: 73; Ident.: 83.3%	Bat coronavirus 512/2005 (BtCoV) (BtCoV/512/2005)
A0A0U1WHB7	E-value: 0.61; Score: 72; Ident.: 83.3%	BtMf-AlphaCoV/JX2012
A0A0U1WHB3	E-value: 0.61; Score: 72; Ident.: 83.3%	BtMf-AlphaCoV/AH2011
A0A2H4MXZ6	E-value: 1.2; Score: 70; Ident.: 83.3%	Shrew coronavirus

A0A1L7HJ82	E-value: 1.2; Score: 70; Ident.: 83.3%	Lucheng Rn rat coronavirus
A0A172AZS6	E-value: 1.7; Score: 69; Ident.: 75.0%	Ferret coronavirus
A0A059VFK8	E-value: 1.7; Score: 69; Ident.: 83.3%	Porcine epidemic diarrhea virus
A0A0U1UZD0	E-value: 1.7; Score: 69; Ident.: 100.0%	BtNv-AlphaCoV/SC2013
P0DTC2	E-value: 1.7; Score: 69; Ident.: 83.3%	Severe acute respiratory syndrome coronavirus 2 (2019-nCoV) (SARS-CoV-2)
U5WI05	E-value: 1.7; Score: 69; Ident.: 83.3%	Bat SARS-like coronavirus WIV1
P59594	E-value: 1.7; Score: 69; Ident.: 83.3%	Severe acute respiratory syndrome coronavirus (SARS-CoV)
Q6UZF4	E-value: 1.7; Score: 69; Ident.: 83.3%	SARS coronavirus PUMC02
Q6UZF0	E-value: 1.7; Score: 69; Ident.: 83.3%	SARS coronavirus PUMC03
Q5DIC5	E-value: 1.7; Score: 69; Ident.: 83.3%	SARS coronavirus WH20
H9BR17	E-value: 1.7; Score: 69; Ident.: 83.3%	Night heron coronavirus HKU19
Q3LZX1	E-value: 1.7; Score: 69; Ident.: 83.3%	Bat coronavirus HKU3 (BtCoV) (SARS-like coronavirus HKU3)
Q3I5J5	E-value: 1.7; Score: 69; Ident.: 83.3%	Bat coronavirus Rp3/2004 (BtCoV/Rp3/2004) (SARS-like coronavirus Rp3)
Q0Q475	E-value: 1.7; Score: 69; Ident.: 83.3%	Bat coronavirus 279/2005 (BtCoV) (BtCoV/279/2005)
A0A0U1WHI2	E-value: 1.7; Score: 69; Ident.: 83.3%	BtRs-BetaCoV/HuB2013
R9QTH3	E-value: 1.7; Score: 69; Ident.: 83.3%	Bat coronavirus Cp/Yunnan2011
R9QTA0	E-value: 1.7; Score: 69; Ident.: 83.3%	Bat coronavirus Rp/Shaanxi2011
I1VWF5	E-value: 1.7; Score: 69; Ident.: 83.3%	Alpaca respiratory coronavirus

G7KFT7	E-value: 1.7; Score: 69; Ident.: 90.0%	Medicago truncatula (Barrel medic) (Medicago tribuloides)
A0A2K3PFZ1	E-value: 1.7; Score: 69; Ident.: 90.0%	Trifolium pratense (Red clover)
Q6Q1S2	E-value: 2.5; Score: 68; Ident.: 83.3%	Human coronavirus NL63 (HCoV-NL63)
A0A150H2E9	E-value: 2.5; Score: 68; Ident.: 75.0%	Gonium pectorale (Green alga)
A0A5N4A2Q1	E-value: 3.5; Score: 67; Ident.: 100.0%	Photinus pyralis (Common eastern firefly) (Lampyris pyralis)
A0A0U1WJW2	E-value: 3.5; Score: 67; Ident.: 81.8%	BtRf-AlphaCoV/HuB2013
A0A0U1UZ37	E-value: 3.5; Score: 67; Ident.: 81.8%	Alphacoronavirus BtMs-AlphaCoV/GS2013
H9BR25	E-value: 3.5; Score: 67; Ident.: 83.3%	Wigeon coronavirus HKU20
A0A240FW18	E-value: 3.5; Score: 67; Ident.: 75.0%	Wencheng Sm shrew coronavirus
Q5MQD0	E-value: 7.0; Score: 65; Ident.: 83.3%	Human coronavirus HKU1 (isolate N1) (HCoV-HKU1)
A0A140H1H1	E-value: 7.0; Score: 65; Ident.: 83.3%	Human coronavirus HKU1 (HCoV-HKU1)
K4JZP8	E-value: 7.0; Score: 65; Ident.: 81.8%	Hipposideros bat coronavirus HKU10
A0A2G2XFP5	E-value: 7.0; Score: 65; Ident.: 75.0%	Capsicum baccatum (Peruvian pepper)
A0A1B3Q5W5	E-value: 9.9; Score: 64; Ident.: 75.0%	Rousettus bat coronavirus
A3EXJ0	E-value: 9.9; Score: 64; Ident.: 75.0%	Bat coronavirus HKU9-4
A0A0U1WJW4	E-value: 9.9; Score: 64; Ident.: 75.0%	BtRf-AlphaCoV/YN2012
A8JNZ2	E-value: 9.9; Score: 64; Ident.: 75.0%	Rhinolophus bat coronavirus HKU2
V7BAS0	E-value: 10.0; Score: 64; Ident.: 90.0%	Phaseolus vulgaris (Kidney bean) (French bean)

A0A662ZKU3	E-value: 10.0; Score: 64; Ident.: 81.8%	Ruminobacter amylophilus

a A blastp search at https://blast.ncbi.nlm.nih.gov/Blast.cgi?PAGE=Proteins produced >5000 hits with identity ≥ 83.3% and 795 hits with 100% identity, but with considerable duplication

[b] BLAST E-value is the number of expected hits of similar quality (score) that could be found just by chance, Score: BLAST score of alignment, Identity: percentage of sequence identity

Table S2. Parameters extracted from the fitting of the SAXS curves in Figure 2c, using a Gaussian coil form factor.

c / parameters*	1 wt% (native)	0.5 wt% (native)
R_g/ Å	7	7.3
ρ_p / a.u.	5	3.5
m_d/ a.u.	0.6	0.6
ρ_{so}/ a.u.	-0.2	-0.2
A/ kDa	0.25	0.2
q_0/ $Å^{-1}$	1.1	0.95
w/ $Å^{-1}$	0.8	0.84
c/ kDa	0.16	0.18

Key: Gaussian Coil Form Factor: radius of gyration, R_g; scattering length of chain, ρ_p; mass density of chain, m_d; scattering length density of solvent, ρ_{so}. **Gaussian Structure Factor:** Amplitude, A; position, q_0; width, w; constant background, c.

*SAXS intensity data is in kDa, and can be converted to cm^{-1} using a conversion factor 0.000802551 which also puts the scattering contrast into units of cm^{-2}.

Table S3. Parameters extracted from the fitting of the SAXS data in Figure 5 and Figure 6f, using the form factors for Gaussian bilayer or monomers (Gaussian coils).

pH (1 wt%) / parameters	pH 4	pH 4.4	pH 5	pH 6	pH 7	pH 12
$N/\ cm^{-1}$	1×10^{-7}	1×10^{-7}	1×10^{-8}	6×10^{-8}	--	--
$2z_H\pm\Delta_{2zH}$ / Å	25 ± 10	25 ± 10	18 ± 15	23 ± 13	--	--
σ_H / Å	14	14	16	14.5	--	--
$\eta_H\ /\ cm^{-2}$	1.2×10^{-3}	0.6×10^{-3}	1.2×10^{-3}	6.5×10^{-4}	--	--
σ_C / Å	9	9	12	10.9	--	--
$\eta_C\ /\ cm^{-2}$	-1×10^{-3}	-0.5×10^{-3}	-1.5×10^{-3}	-1×10^{-3}	--	--
R_g/ Å	--	--	--	--	8	8
$\rho_p\ /\ cm^{-2}$	--	--	--	--	0.1	0.085
$m_d/\ g\ cm^{-3}$	--	--	--	--	0.6	0.6
$\rho_{so}/\ cm^{-2}$	--	--	--	--	-0.2	-0.2
D	550	720	600	700		
a	1×10^{-7}	1×10^{-7}	5×10^{-4}	7×10^{-7}	--	--
b	3.4	3.4	3.8	3.2	--	--
c	1.5×10^{-3}	1.5×10^{-4}	5×10^{-4}	2×10^{-3}	6×10^{-4}	2×10^{-3}

Key: Gaussian Bilayer: Scale factor, N; Gaussian half-width at half-maximum for polydispersity, Δ_{2zH}; inter-head group thicknesses, $2z_H$; Gaussian half-width for outer layer surface, σ_H; electron density 'headgroup' (outer surface layer), η_H; Gaussian half-width for inner layer, σ_C; relative electron density for inner layer, η_C; diameter D. The background is described by $(a/q^b) + c$. **Gaussian Coil Form Factor:** Radius of gyration, R_g; scattering length of chain, ρ_p; mass density of chain, m_d; scattering length density of solvent, $\rho_{so;}$ background, c.

Table S4. Parameters extracted from the fitting of the SAXS data in Figure 9, using the form factor of a Gaussian bilayer.*

c (pH 4) / parameters	0.3 wt%	0.4 wt%	0.5 wt %	0.6 wt%	0.7 wt%	0.8 wt%	1 wt%
N/ cm^{-1}	1×10^{-7}	1×10^{-7}	1×10^{-7}	1×10^{-7}	1×10^{-7}	1×10^{-7}	1×10^{-7}
$2z_H\pm\Delta_{2zH}$ / Å	30 ± 10	20 ± 10	21 ± 10	22 ± 10	18 ± 12	21 ± 10	25 ± 10
σ_H / Å	13	13.8	13	13.8	12.5	13.8	14
η_H / cm^{-2}	3.5×10^{-4}	7×10^{-4}	8×10^{-4}	8.8×10^{-4}	9×10^{-4}	1×10^{-3}	1.2×10^{-3}
σ_C / Å	10	10	10	10	10	10	9
η_C / cm^{-2}	-3.5×10^{-4}	-1×10^{-3}	-8×10^{-4}	-1×10^{-3}	-1×10^{-3}	-1×10^{-3}	-1×10^{-3}
D	600	710	600	650	650	600	550
a	--	5×10^{-7}	--	--	--	--	1×10^{-7}
b	--	2.8	--	--	--	--	3.4
c	1×10^{-3}	8×10^{-4}	1.2×10^{-3}	1.3×10^{-3}	1.5×10^{-3}	1.8×10^{-3}	1.5×10^{-3}

Key: Scale factor, N; Gaussian half-width at half-maximum for polydispersity Δ_{2zH}; inter-head group thicknesses, $2z_H$; Gaussian half-width for outer layer surface, σ_H; electron density for 'headgroup' (outer surface layer), η_H; Gaussian half-width for inner layer, σ_C; relative electron density for inner layer, η_C; diameter, D. The background is described by $(a/q^b) + c$.

*Data for 1 wt% is the same as in Table S3 and appears here for comparative purposes.

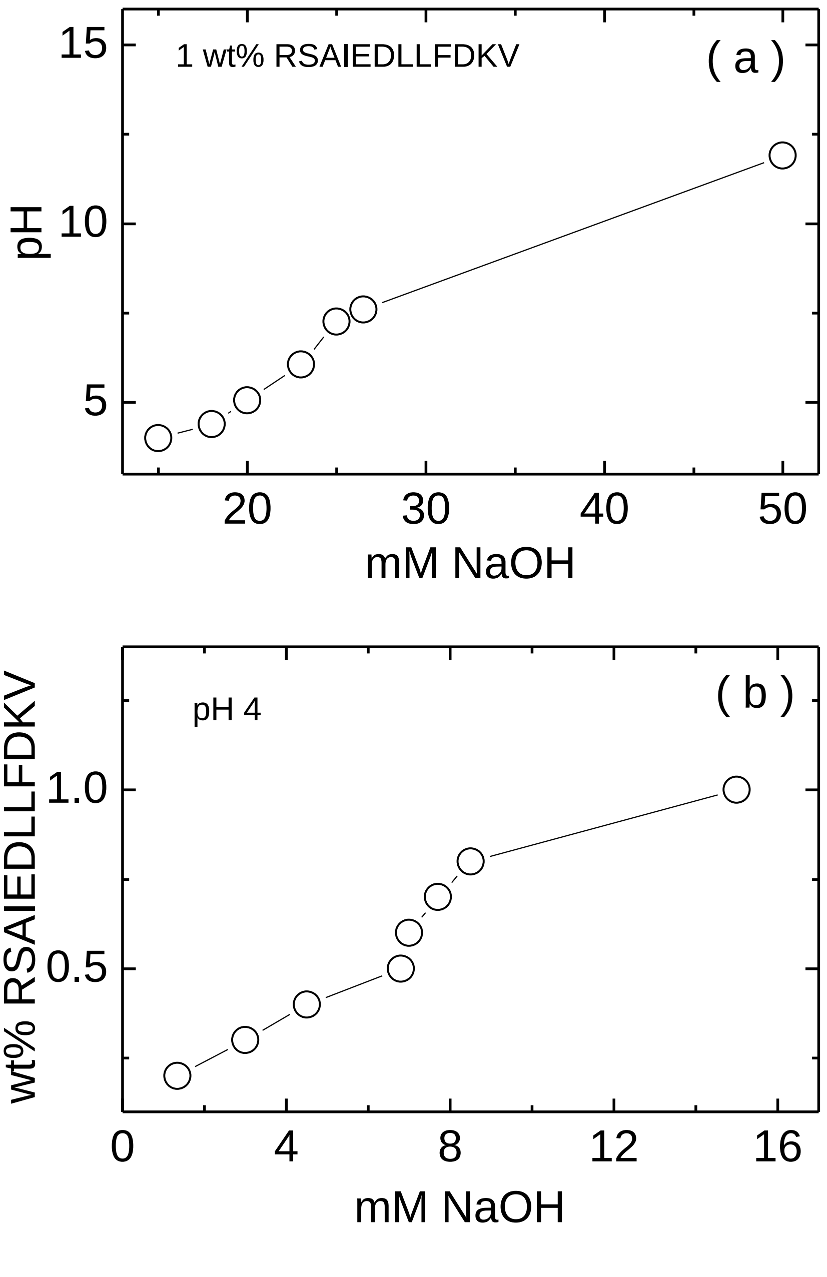

Figure S1. (a) Concentration of NaOH solutions used as a solvent to obtain different pH solutions at 1 wt% peptide. (b) Concentration of NaOH solutions used to obtain different peptide concentrations at pH 4.

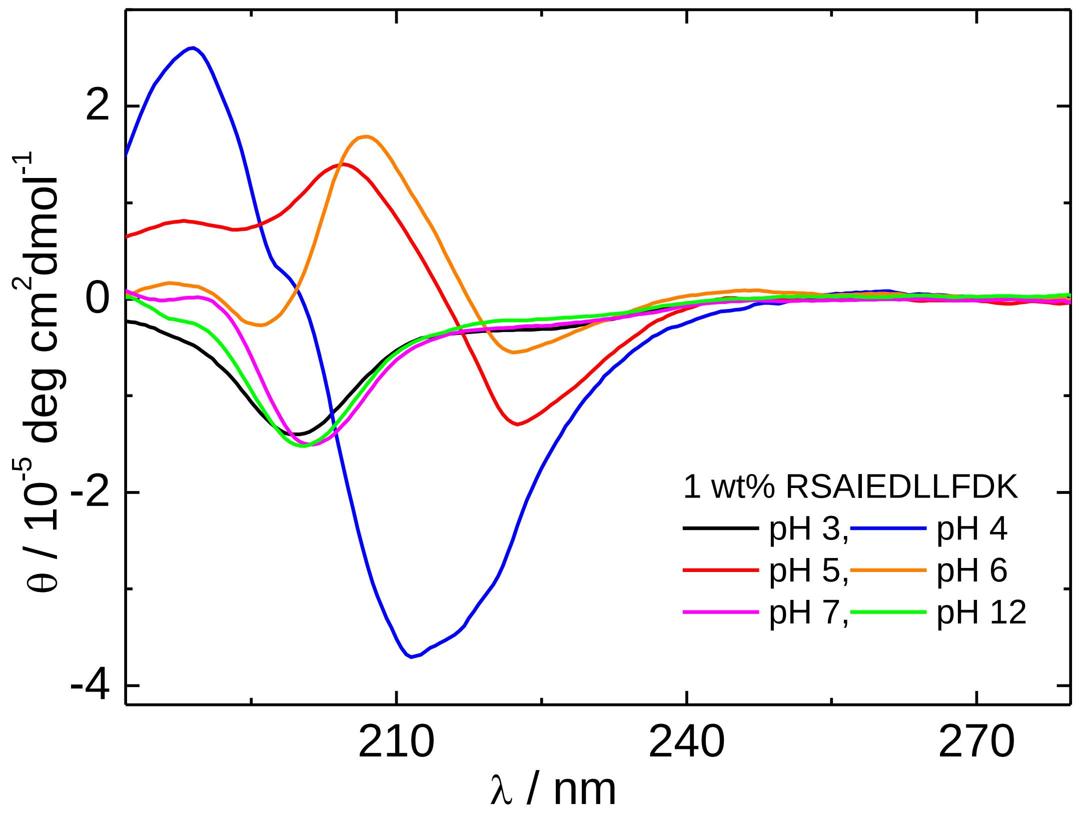

Figure S2. CD spectra for 1 wt% RSAIEDLLFDK as a function of pH (pH 3-12; solutions dissolved in pure water are native at pH 3).

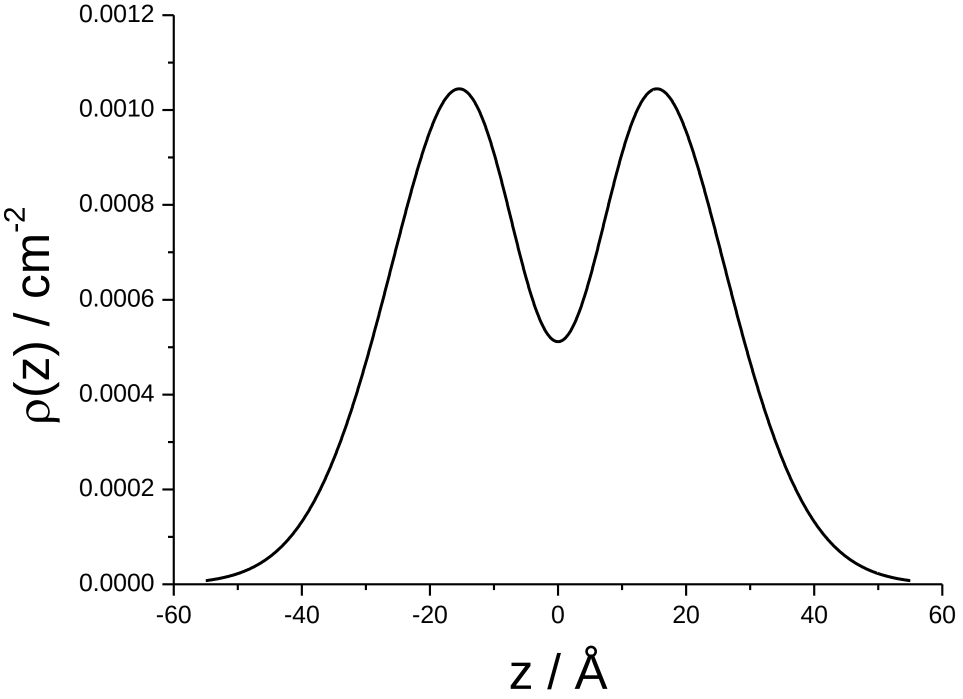

Figure S3. Electron density profile calculated using the parameters for 1 wt% RSAIEDLLFDKV at pH 4 from Table S3.

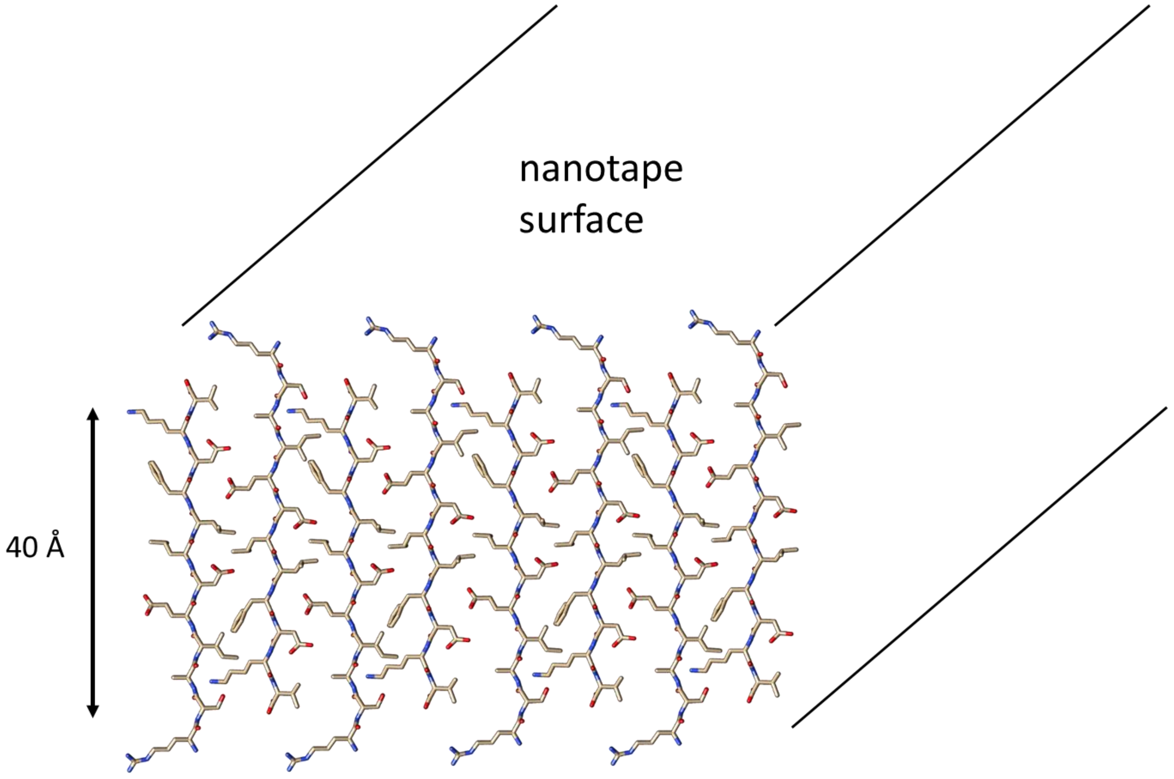

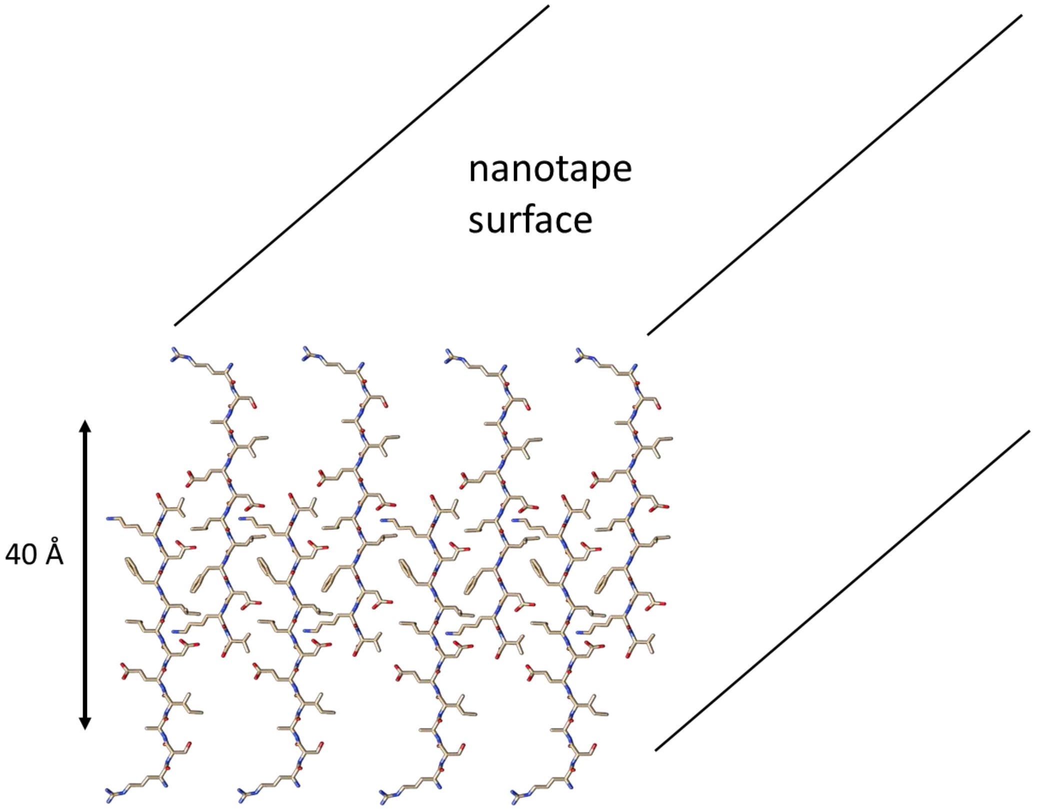

Figure S4. Two possible plausible models for molecular packing in the arginine-coated bilayer nanotapes, based on parameters from SAXS along with analysis of likely hydrophobic core residues.

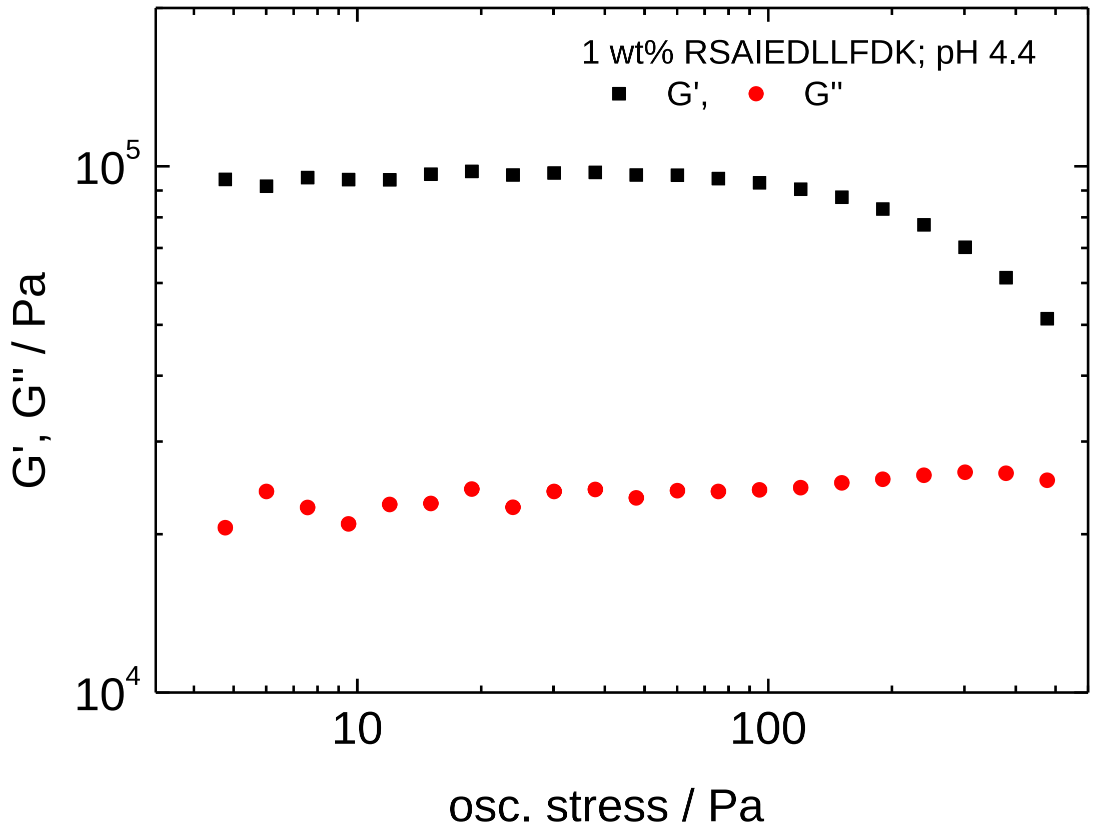

Figure S5. Shear moduli *versus* stress, showing linear viscoelastic regime for the 1 wt% RSAIEDLLFDK pH 4.4 gel.

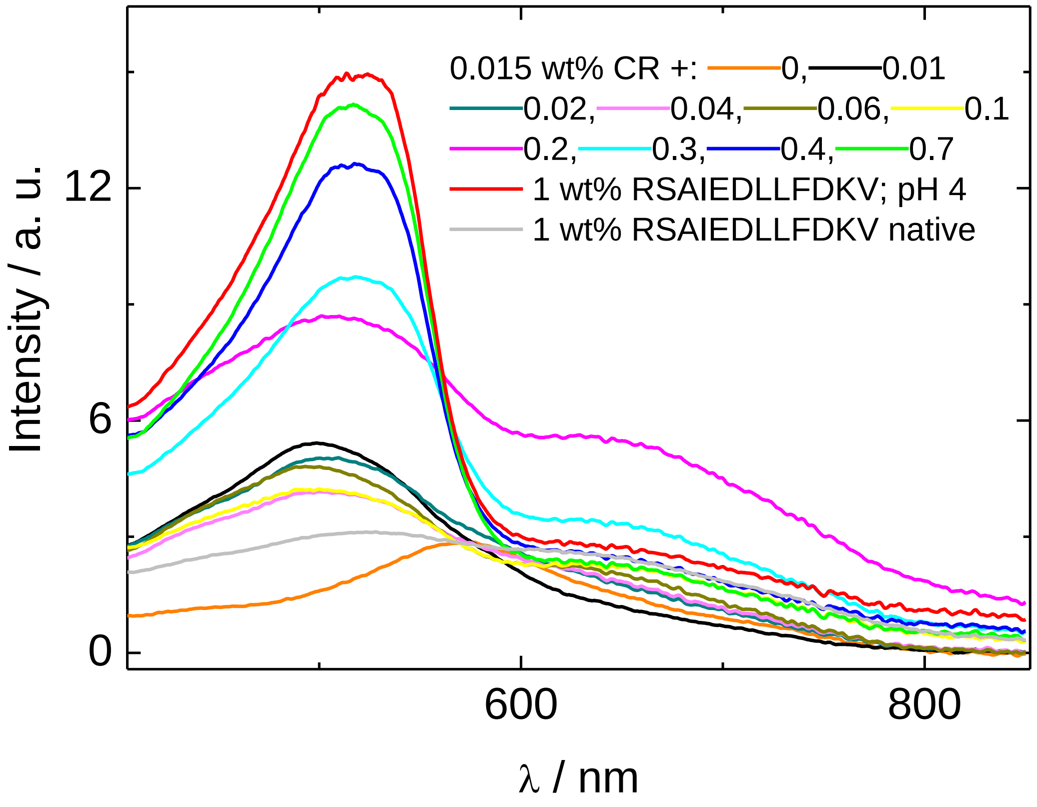

Figure S6. UV-vis spectra for peptide solutions (pH 4) containing 0.015 wt% Congo red. The UV-vis for a native solution of 1 wt% peptide (pH 3) stained with Congo red is also displayed for comparative purposes.

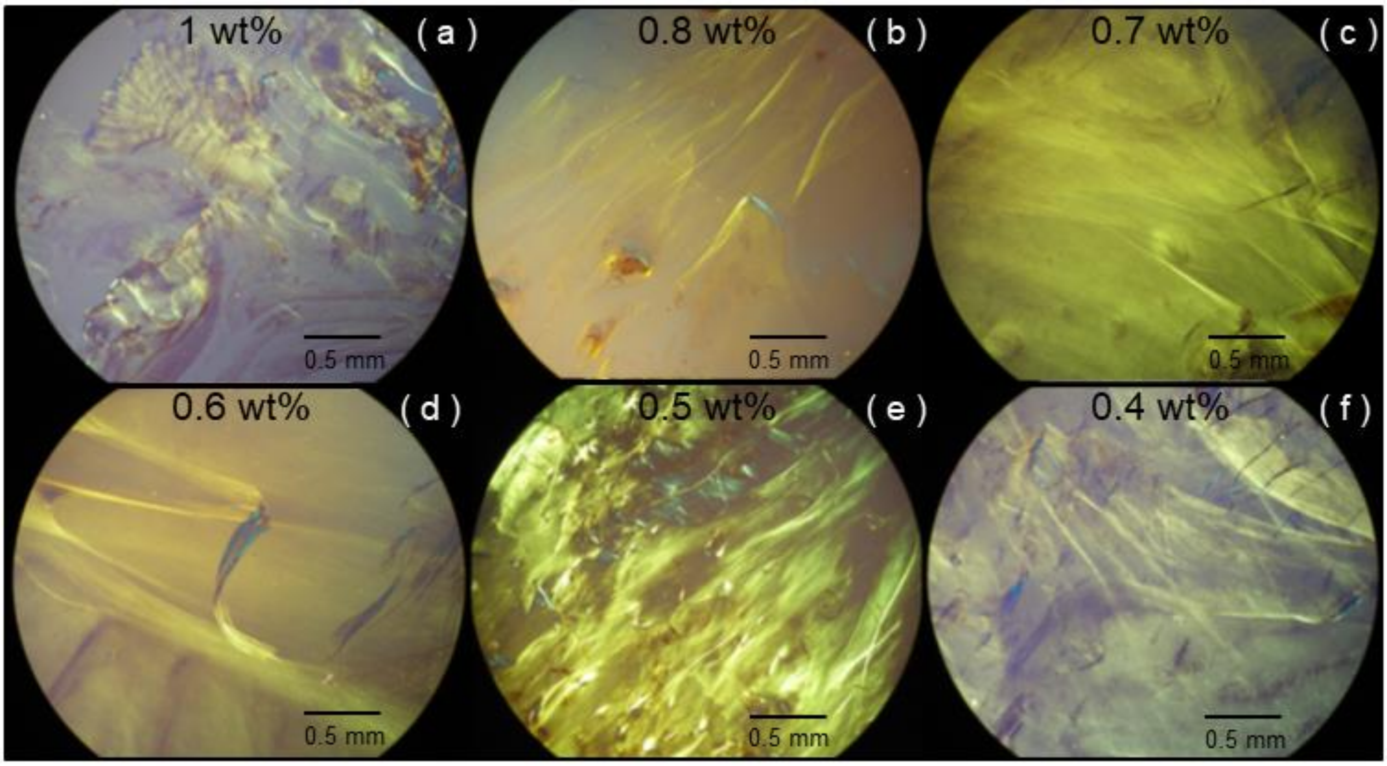

Figure S7. Polarized optical microscopy images showing Congo red birefringence measured for 1 - 0.4 wt% RSAIEDLLFDK at pH 4.

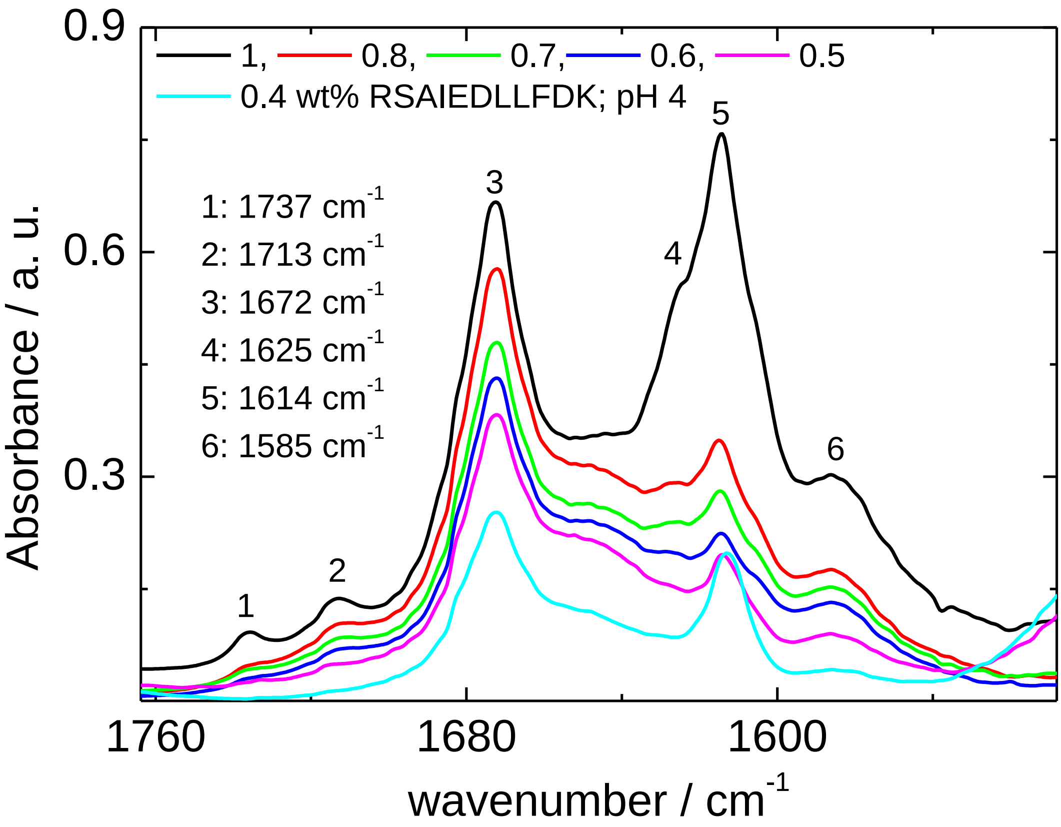

Figure S8. FTIR spectra for 1- 0.4 wt% RSAIEDLLFDKV solutions (pH 4).

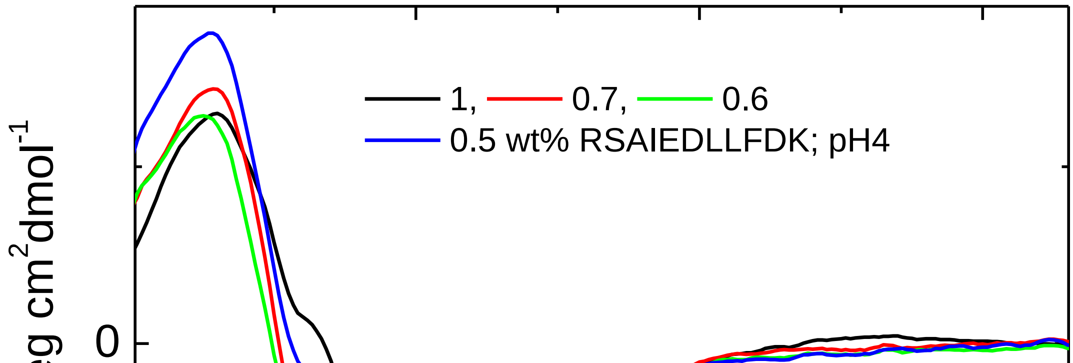

Figure S9. CD spectra for solutions as a function of the concentration (pH 4).

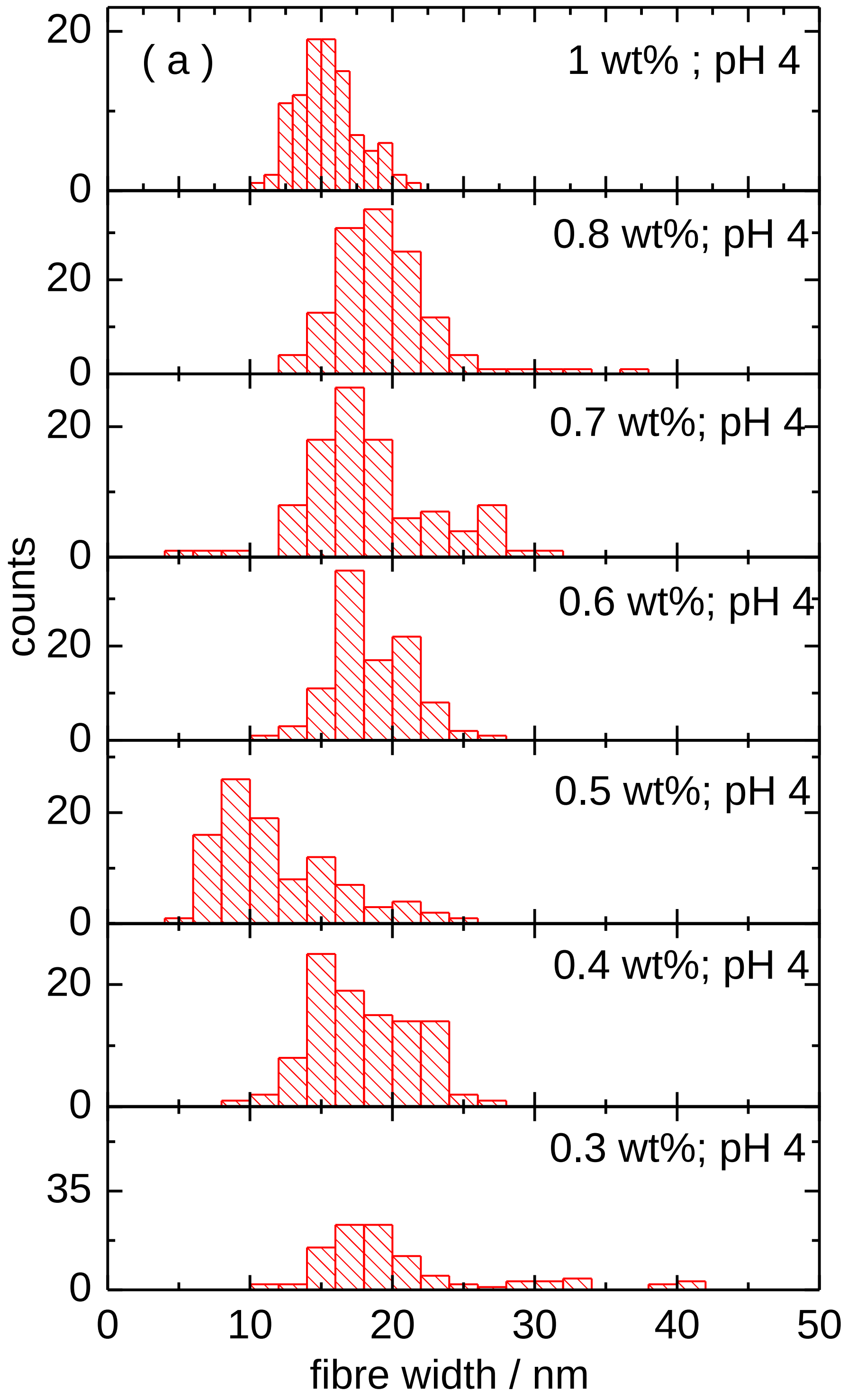
(a)
1 wt% ; pH 4
0.8 wt%; pH 4
0.7 wt%; pH 4
0.6 wt%; pH 4
0.5 wt%; pH 4
0.4 wt%; pH 4
0.3 wt%; pH 4
counts
fibre width / nm
0
10
20
30
40
50
35

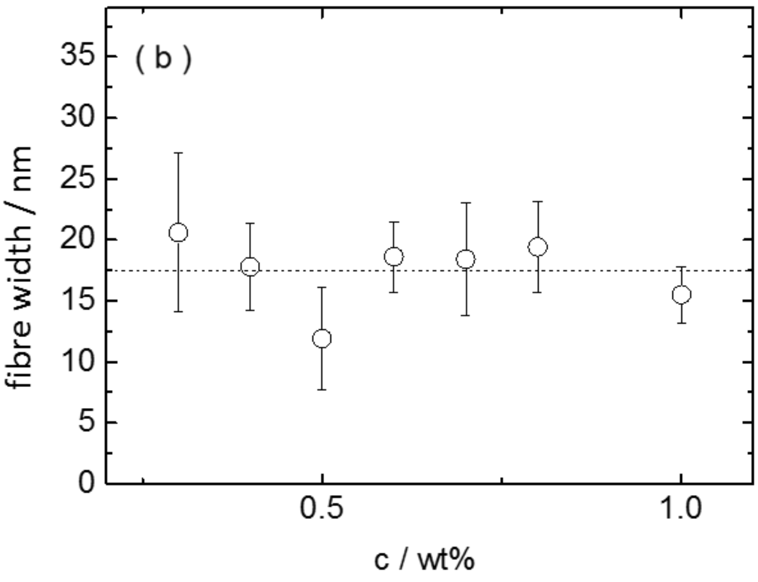

Figure S10. (a) Histograms showing the distribution of fibre widths measured from TEM images in Figure 8, (b) Average fibre width as a function of the concentration calculated using the values plotted in (a).

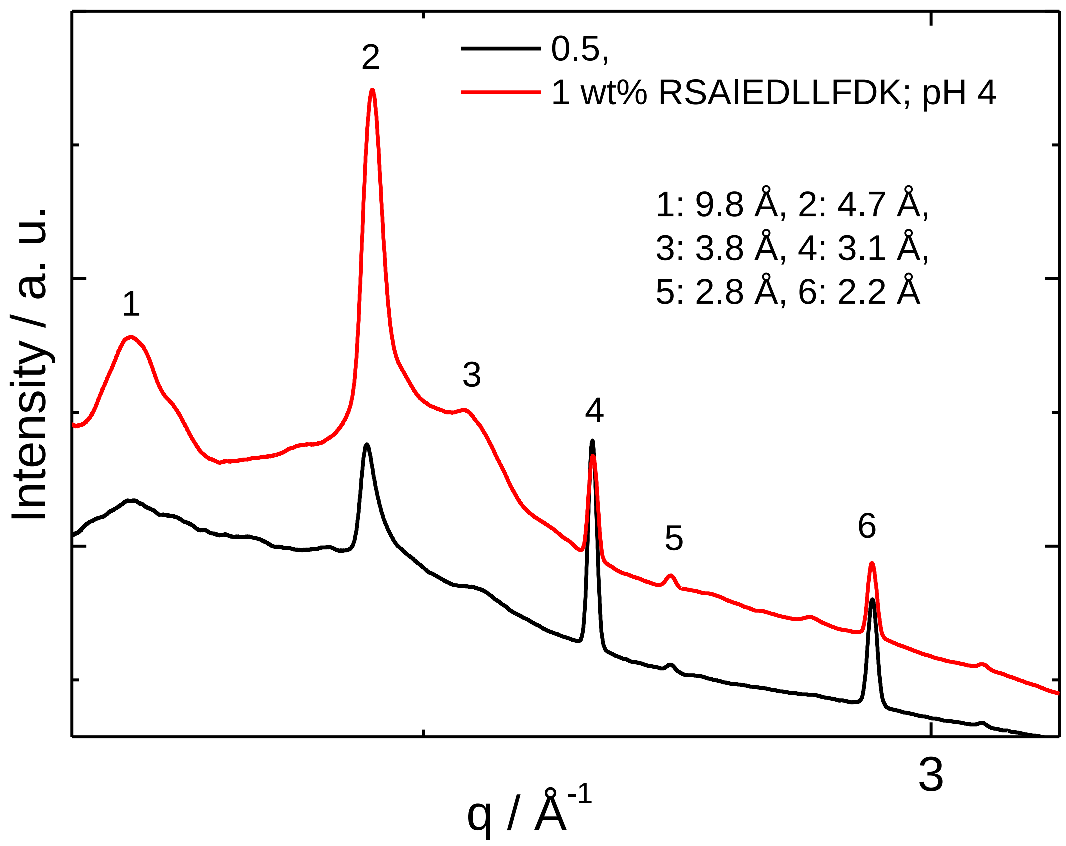

Figure S11. XRD profiles for peptide stalks dried from peptide solutions at pH 4.
